# Reorganizing heterogeneous information from host–microbe interaction reveals innate associations among samples

**DOI:** 10.1002/qub2.25

**Published:** 2023-11-29

**Authors:** Hongfei Cui

**Affiliations:** ^1^ Department of Management Science and Engineering School of Economics and Management University of Science and Technology Beijing Beijing China

**Keywords:** 16S rRNA, graph embedding, heterogeneous information network, microbiome

## Abstract

The information on host–microbe interactions contained in the operational taxonomic unit (OTU) abundance table can serve as a clue to understanding the biological traits of OTUs and samples. Some studies have inferred the taxonomies or functions of OTUs by constructing co‐occurrence networks, but co‐occurrence networks can only encompass a small fraction of all OTUs due to the high sparsity of the OTU table. There is a lack of studies that intensively explore and use the information on sample‐OTU interactions. This study constructed a sample‐OTU heterogeneous information network and represented the nodes in the network through the heterogeneous graph embedding method to form the OTU space and sample space. Taking advantage of the represented OTU and sample vectors combined with the original OTU abundance information, an Integrated Model of Embedded Taxonomies and Abundance (IMETA) was proposed for predicting sample attributes, such as phenotypes and individual diet habits. Both the OTU space and sample space contain reasonable biological or medical semantic information, and the IMETA using embedded OTU and sample vectors can have stable and good performance in the sample classification tasks. This suggests that the embedding representation based on the sample‐OTU heterogeneous information network can provide more useful information for understanding microbiome samples. This study conducted quantified representations of the biological characteristics within the OTUs and samples, which is a good attempt to increase the utilization rate of information in the OTU abundance table, and it promotes a deeper understanding of the underlying knowledge of human microbiome.

## 1 INTRODUCTION

Microbes are the most abundant and widely distributed residents on earth [1]. They perform crucial functions in the natural ecosystems they inhabit or in the well‐being of their hosts, via forming microbiota and various complex interactions with their niches [2, 3]. Sequencing of 16S rRNA, as a fast and low‐cost method with high sample throughput, provides us with many opportunities to dig into the associations between the structures of microbial communities and characteristics of niches [4]. A very straightforward idea for analyzing 16S rRNA data is to compare the samples based on proper features and then associate the identified shifts or variations with specified sample attributes, such as environmental differences or host health states.

In the analysis of 16S rRNA samples, one of the most commonly used processes is the direct use of operational taxonomic unit (OTU) abundance vectors to represent samples for the subsequent research steps. These steps often treat OTUs as independent features, such as calculating beta diversity between samples [5, 6], unsupervised clustering [7, 8], visualization [9, 10], and classification according to specific sample characteristics [11]. The above approach to utilizing features is very straightforward and can help unearth a great deal of instructive information. However, there are still some aspects that require further improvement. On the one hand, treating OTUs as independent features in the analysis ignores a lot of important information, such as the correlation between OTUs and their biological functions. This correlation is closely related to the understanding of sample biological characteristics and may greatly facilitate the comparison between samples. On the other hand, OTU matrixes are often very sparse, with very few overlapping features between samples. There are numerous sample‐specific OTUs, and some of them, despite appearing in different samples, maintain intricate and complex connections. While these OTUs often exhibit distinct nucleic acid sequences for evolutionary reasons, they may share similar ecological functions. This inherent connection is challenging to directly capture through sparse OTU abundance vectors.

The analysis of the OTU co‐occurrence relationships based on the variation of OTU abundance between samples, such as constructing a co‐occurrence network by calculating the Spearman’s correlation coefficients between OTUs, can be used to infer the taxonomy [12] or functions [13] of OTUs. Software developed on the above basis, such as TACO [12] and HOPE [13], has achieved great results. Nevertheless, the number of OTUs that can be considered to have co‐occurrence relationships with other OTUs, and therefore understood through co‐occurrence networks, is very limited. For example, in the dataset of soil microbes used in a study using TACO, only 572 OTUs, among a total of 13,490, were constructed in the co‐occurrence network (Spearman’s correlation coefficient value >0.5, *p*‐value <0.05). As a result, the information existing in the vast majority of OTUs cannot be fully utilized. Meanwhile, OTU matrixes are becoming sparser as the number of considered samples are increasing, and this creates statistical difficulties in recognizing co‐occurrence relations. For example, in the American Gut Project (AGP [14]), there were over 15,000 samples of gut microbes and over 30,000 OTUs, of which only 5000 OTUs appeared in more than 100 samples. Thus, it is difficult to obtain reliable OTU similarity information by traditional correlation calculation. The difficulty in constructing co‐occurrence networks due to excessively sparse abundance matrixes has been noticed in other fields of study. For example, in single‐cell sequencing, the scRNA‐seq gene expression data are often much sparser than traditional RNA‐seq data. To solve this problem, scholars have tried various methods, such as the information theory [15] used by PIDC, the tree‐based ensemble methods of GENIE3 [16], and the statistical network modeling [17] of scLink. Although these methods have inspired solutions to the problem of extremely sparse OTU abundance matrixes, they have not solved the problem that most OTUs cannot be organized into co‐occurrence networks.

From another perspective, OTU abundance matrixes naturally contain interaction information regarding microorganisms and their environments, which can be used to reveal the functional characteristics of OTUs to some extent. The variation of relative abundance of each OTU in the microbiota of different samples shows the degree of adaptation of that OTU in environments. The variation, to some extent, reflects the effects of the interaction between hosts and microbes and can be represented by constructing a heterogeneous information network of sample‐OTUs. The nodes in the network can be expressed as quantified low‐dimensional vectors with semantic information of microbe biological characteristics. Graph embedding methods have been widely used in microbe‐disease [18, 19, 20, 21] or microbe‐drug [22, 23] association prediction. Knowledge of the networks embedded in these studies is often based on public databases describing the relationships between microbes with diseases or drugs, such as Human Virulence Factor Database [24], Microbe‐Disease Association Database (HMDAD) [25], and Microbe‐Drug Association Database [26]. As the entries in these databases are mostly experimentally validated or collected from published literature, the number of microbes involved is limited, and they cannot cover all OTUs in 16S rRNA sequencing samples. Direct extraction of the biological semantic representation of OTUs from the sample‐OTU interactive network is still subject to lack of study.

Based on the above idea, this study used the OTU abundance matrix in AGP [14], containing over 10,000 samples and 20,000 OTUs, to establish a sample‐OTU heterogeneous information network based on the OTU abundance variation among samples. MetaPath2Vec was used to embed OTU nodes and sample nodes, and OTU space and sample space were formed. In the OTU space visualized by t‐SNE, OTUs with medium and high abundance and those with low abundance formed two horn‐shaped regions. Taxonomies were not the main factor affecting the positions of OTUs in the space. OTUs in the same regions tend to have similar biological functions and ecological niches. In the visualized sample space, the samples showed a twisted‐rope‐like distribution (see Section 2.3), with the OTU richness gradually increasing from the left to the right. According to the study of 134 sample attributes of interest, from a global perspective of space, there were no attributes that could dominate the sample distribution, while it could be found that many attributes tended to lead to local aggregation of samples. Gut microbiota of all healthy individuals tends to be alike, but for most diseases every patient has a unique microbiota. From the analysis on local aggregation, this study found some phenotypes and individual characteristics that may have association with gut microbiota but there is a lack of scientific research on factors such as vivid dreams, flu vaccination, level of education. This study established an Integrated Model of Embedded Taxonomies and Abundance (IMETA) utilizing information from both the OTU space and sample space to predict phenotype or individual characteristic values. Compared with baseline methods that use only OTU abundance or embedded sample vector, IMETA can obtain a superior and stable performance. This study shows that the reorganization of host–microbe interaction information using heterogeneous information network embedding can provide us with a deeper understanding of OTUs’ biological functions and underlying physiological mechanisms in host samples.

## 2 RESULTS

### 2.1 Data description and research design

The sample attributes and OTUs of fecal samples from the AGP are used as the objects of this study. Human gut microbiota has a complex structure and many influencing factors. Many aspects of the host, including disease states (e.g., inflammatory bowel disease [27, 28], type 2 diabetes [29, 30], and cancer [31, 32]), lifestyle habits (e.g., smoking [33, 34], drinking [35, 36], and dietary habits [37, 38]), and medications [39, 40], etc., all have potential associations with changes in the structure of gut microbial communities. However, it is difficult to classify them precisely simply through the abundance vectors of microbes, and this is worth exploring. The AGP, as one of the predecessors of The Microsetta Initiative, a worldwide collaborative initiative, collected large numbers of 16S rRNA sequencing samples, with various sample attributes recorded as metadata. It performed OTU clustering and taxonomy annotation on the sequencing data of the samples, developing an OTU abundance table with metadata that can be a good basis of research for scholars.

The latest version of the OTU table relating to AGP fecal samples was downloaded. After data preprocessing (see Section 3.1), 11,184 samples with a depth of more than 10,000 OTUs were screened, and there were a total of 23,464 OTUs that appeared at least once in these samples. As precise taxonomy annotation can hardly be performed on 16S rRNA sequenced OTUs, the annotation of most OTUs only reached order or family level. Over half of OTUs did not have annotation at genus level, and this proportion exceeded 90% at species level (Table 1). To investigate the relationship between OTUs and sample attributes, we selected 134 attributes of interest from the metadata of gut microbial samples of the AGP (Section 3.1), and, after combining the synonymous options of these attributes, possible values of each attribute is shown in Table S1.

**TABLE 1:** Stats of OTU taxonomy annotation.

Taxonomy level	Number of taxa	Number of unannotated OTUs (percentage)
Phylum	55	1 (0.004%)
Class	170	103 (0.439%)
Order	356	755 (3.218%)
Family	632	5144 (21.923%)
Genus	1567	13,746 (58.583%)
Species	2086	21,684 (92.414%)

Abbreviation: OTU, operational taxonomic unit.

**FIGURE 1:**
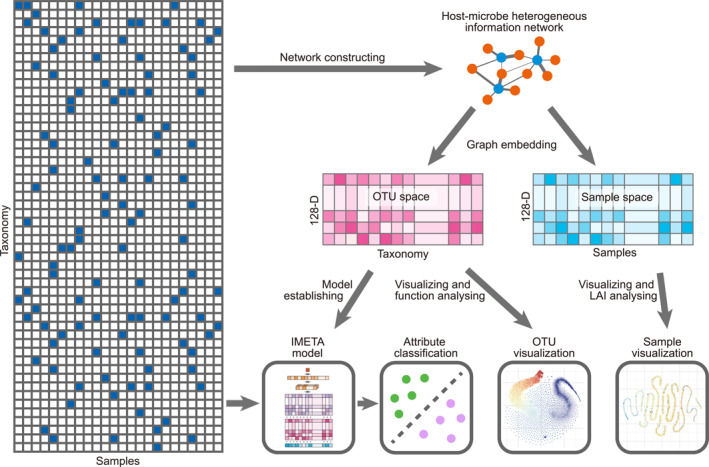
The overall pipeline of the proposed method in this study.

Figure 1 shows the pipeline of the proposed method in this study. The OTU abundance table presents the interaction information between microbes and their associated samples. It was reconstructed into a host–microbe heterogeneous information network, and a heterogeneous network embedding model was applied to generate the representations of microbes and sample nodes. For microbes (i.e., represented as OTUs in calculations), on the one hand, the biological semantic meanings of these OTU embedding vectors could be confirmed through visualization and functional analysis. On the other hand, these representation vectors containing functional information were further combined with the original OTU abundance information of the samples, to predict whether a sample contained a certain attribute. For samples, in addition to visualizing these embedding vectors to investigate the overall character of sample attributes, this study also defined a Local Aggregation Index (LAI, see Section 3.3.2) in the sample space to evaluate the degree to which sample attributes were associated with the microbial community structure.

### 2.2 Characteristics of the OTU space

#### 2.2.1 Overview of the OTU distribution

**FIGURE 2:**
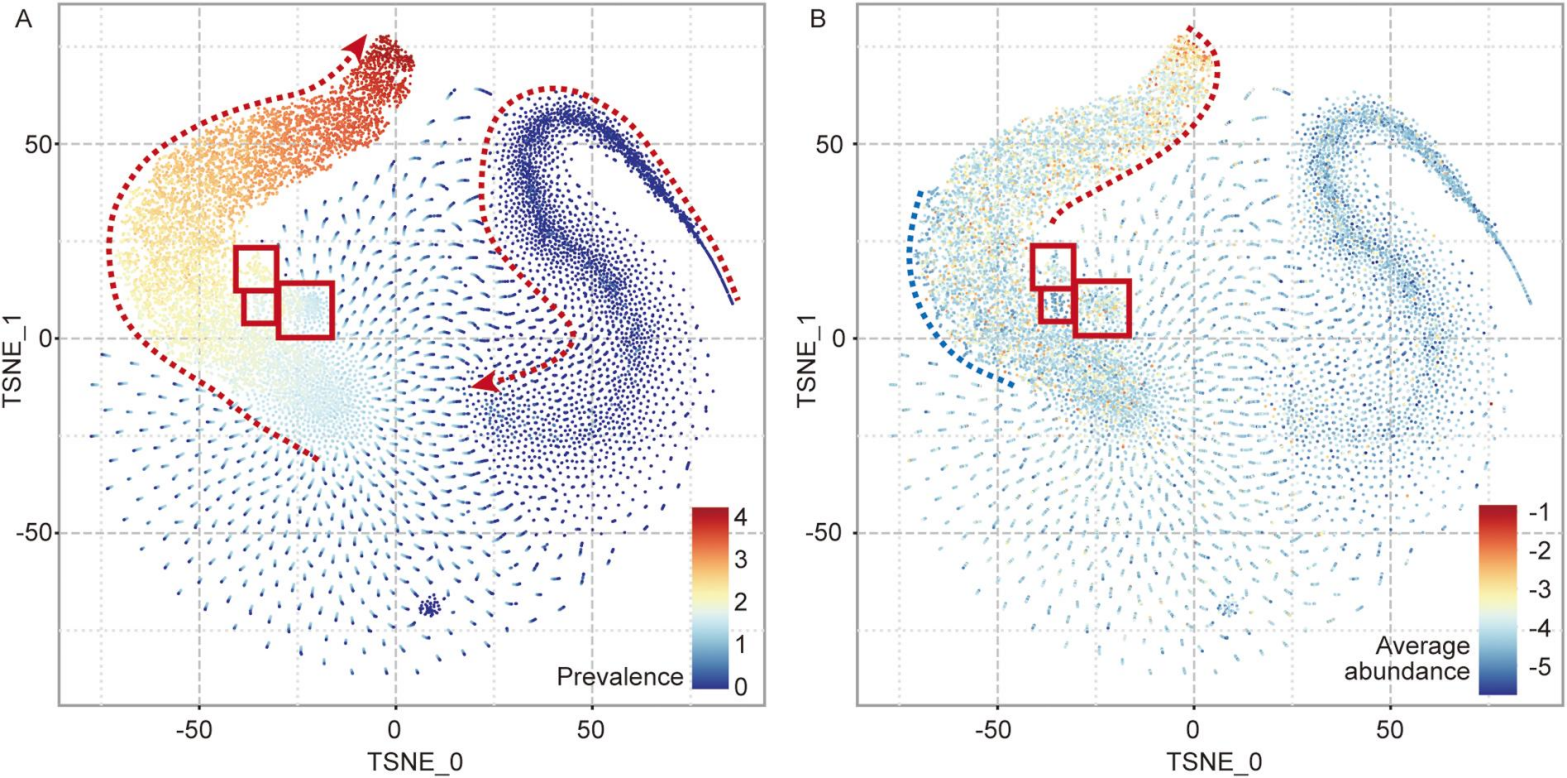
OTU distribution according to (A) prevalence and (B) average abundance. Red dashed line with arrow in panel (A): main change direction of prevalence; red boxes: the three distinct clusters close to the left horn. Red dashed line in panels (B), indicating the high abundance OTUs region. Blue dashed line in panel (B): indicating the low abundance OTUs region. OTU, operational taxonomic unit.

After establishing a sample‐OTU network and embedding network nodes using MetaPath2Vec, the distributed representation of the samples and OTUs were obtained (Section 3.2). Figure 2 shows the distribution of all the 23,464 OTUs that appeared in the 11,184 samples in the OTU space.

It can be seen that OTUs were clustered into two main parts, exhibiting a two‐horn‐shaped distributions in the t‐SNE visualized space (Figure 2A). One part was mainly distributed in the left horn region (TSNE0 < 5), and the other part was mainly distributed in the right horn region (TSNE0 > 10). Although we did not emphasize information on prevalence in the construction of the network, we could still see that OTUs were distributed in the space along the axial direction of a horn (Figure 2, red dotted arrow) with a very significant trend. OTUs that appeared in more than 10 samples were generally in the left horn. In particular, samples in the middle and top sections (TSNE1 > 25) generally appeared in more than 1000 samples. In the right horn, the prevalence of OTUs was largely <10, and a large number of OTUs appeared in only one or two samples. The result that these ultra‐rare OTUs are gathered in a distinctly different cluster is a very interesting phenomenon. We analyzed the metapath sequences, based on MetaPath2Vec, and confirmed that all sample nodes and OTU nodes were in the same connected graph. This meant that the situation in which two parts of nodes were embedded in contrasting space regions was not triggered by any defects of network structure, but by the natural characteristics of the microbes. At the same time, many ultra‐rare OTUs which usually appeared in drastically different samples became clustered, suggesting that the network embedding representation method may capture OTU similarities at a higher order, encompassing aspects such as biological functions, environmental adaptability, and more. Such higher order similarities cannot be directly visualized through the OTU abundance table or sample‐OTU network.

The average abundance of OTUs in the samples where they occur, referred to as “average abundance” in this paper, has also been calculated (Section 3.3.1), and showed a different distribution pattern. Overall, OTU abundance in the left horn was higher than that in the right horn, but there were still a number of low‐abundance OTUs distributed in the middle and lower part of the left horn. OTUs with the highest abundance tended to distribute at the tip and the inner side of the left horn (Figure 2B, near the red dashed line), while low‐abundance OTUs tended to cluster at the outer size of the horn (Figure 2B, near the blue dashed line). In addition, there were three small clusters by the side of the left horn (Figure 2A, red boxes), which were significantly different from the surrounding clusters in terms of both position and abundance. Specifically, most OTUs in the lower left cluster were low‐abundance, OTUs with lower prevalence but higher abundance were distributed in the cluster on the right‐hand side, and those distributed in the upper left cluster had higher prevalence and medium abundance.

The distribution of taxonomy in the OTU space was investigated in this study (Supporting Information S1: Supplementary Results and Figures S1–S3). Although OTUs seem to be generally distributed throughout the space, no matter in which level of taxonomy, each taxon still has its own characteristic distribution pattern. OTUs with lower taxonomy levels showed more significant local aggregations and heterogeneity in the spatial distribution, yet OTUs at the same species level would still be widely distributed across the space. For microorganisms, genetic stratifications and phenotypic classification may differ greatly. A slight difference in the living environment or genotypic differences can result in huge changes in a microbial phenotype [41]. The findings of the present study are consistent with this understanding.

#### 2.2.2 Local aggregation of OTU functional characteristics

**FIGURE 3:**
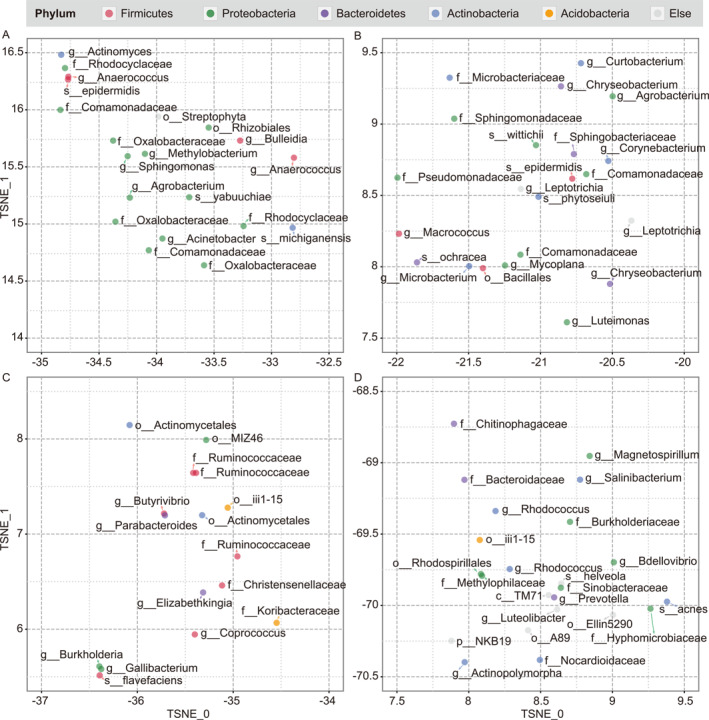
Operational taxonomic units in four example regions (A–D) show different function characteristics.

To investigate whether the locations of OTUs in their space are related to biological functions, this study investigated OTUs in some local regions of the studied space. For example, there were three obvious clusters in the left horn regions of the OTU space (Figure S4A,B). Three example regions were selected (Figure 3A–C) from the three clusters. OTUs in these regions tended to be all related to diets, and OTUs at different locations were associated with different types of diets or corresponding functions.

Figure 3A illustrates the microorganisms in a small region from the upper left cluster from Figure S4. Genera *Methylobacterium* and *Sphingomonas* are dominant and stable microbes in the phyllosphere, together with genus *Acinetobacter*, a famous soil microbe [42, 43]. *Acinetobacter* is also a dominant genus on fresh‐cut vegetables [44]. The genus *Agrobacterium* also exists in the stems and leaves of plants, and it was reported to be found during the culture of the shoot tip of papaya along with *S*. *yabuuchiae*, a species of genus *Sphingomonas* [45]. Besides, in this region, there are several OTUs belonging to family Oxalobacteraceae, which is a common bacterium in water systems and is widely distributed in natural soil, water, and the feces of herbivores [46], and has been detected in fresh‐cut vegetables [44]. It is reasonable to speculate that the microorganisms in this region tend to be derived from vegetable‐based foods.

Microorganisms in Figure 3B (a small region from the right‐hand cluster) tend to come from foods serving as sources of protein, such as dairy products, fish, and meat. In particular, genera *Macrococcus* and *Microbacterium* both widely exist in cow’s milk [47, 48], and genus *Macrococcus* is often detected along with family Comamonadaceae in dairy products [49], fish [50], and meat [51]. Microbacteriaceae (to which *Microbacterium* belongs) and Comamonadaceae are both dominant families in fish, and Microbacteriaceae will replace Comamonadaceae as dominant when the water temperature rises [52], indicating that the two families may have similar ecological niches. Additionally, the species *Staphylococcus epidermidis* [53, 54, 55] and genus *Leptotrichia* [56, 57, 58] are often reported to be found in dairy products, and the genus *Corynebacterium* is considered associated with mastitis in cattle [59, 60, 61].

The region considered in Figure 3C is closer to the left horn, and the predominant microorganisms are commonly associated with the digestion and absorption of cellulose and sugar. Family Ruminococcaceae is widely present in the rumen of ruminants. Along with Ruminococcaceae or its subordinate genus *Ruminococcus*, genera *Butyrivibrio*, *Parabacteroides*, and family Christensenellaceae have all been detected in the rumen of cattle and sheep [62, 63, 64, 65, 66]. *Ruminococcus flavefaciens* is a species from Ruminococcaceae and an important bacterium for decomposition of cellulose [67]. After the intake of insoluble fiber, the abundance of family Ruminococcaceae and genus *Coprococcus* will increase [68], and the abundance of *Coprococcus* increases after the intake of resistant starch [69]. In addition, genus *Burkholderia* is also important in the decomposition of cellulose [70, 71]. *Elizabethkingia* is considered one of the core microbiota in breast milk and the infant gut [72], and it may help infants with initial sugar metabolism.

In addition to the three clusters next to the left horn area, there was a distinct cluster in the fourth quadrant of the OTU space (Figure 3D). The microorganisms in this region are mainly associated with the degradation of pollutants. Both genus *Rhodococcus* and family Methylophilaceae play an important role in plastic degradation [73, 74], sewage treatment [75, 76], and remediation of contaminated agricultural soils [77, 78], and they, along with order Rhodospirillales, can decompose methane and degrade brown coal (a type of low‐quality coal that produces pollution when burned). These organisms have become crucial in microbial enhanced oil recovery technologies; they can degrade energy resources that cannot be easily used in a direct way, to increase oil recovery efficiency [75, 79, 80]. Other microorganisms in this region, such as genus *Burkholderiaceae* and *Bdellovibrio*, are often used in the bioremediation process of oil pollution, antibiotic pollution, and plastic pollution [81, 82, 83].

Although the construction of the OTU space did not rely on any taxonomy or functional annotations, adjacent OTUs still exhibited a high degree of consistency in terms of biological characteristics or ecological niches, which demonstrated the effectiveness of OTU vector representation using the sample‐OTU network.

### 2.3 Phenotypes and individual characteristics in the sample space

#### 2.3.1 Global non‐dominance and local aggregation

**FIGURE 4:**
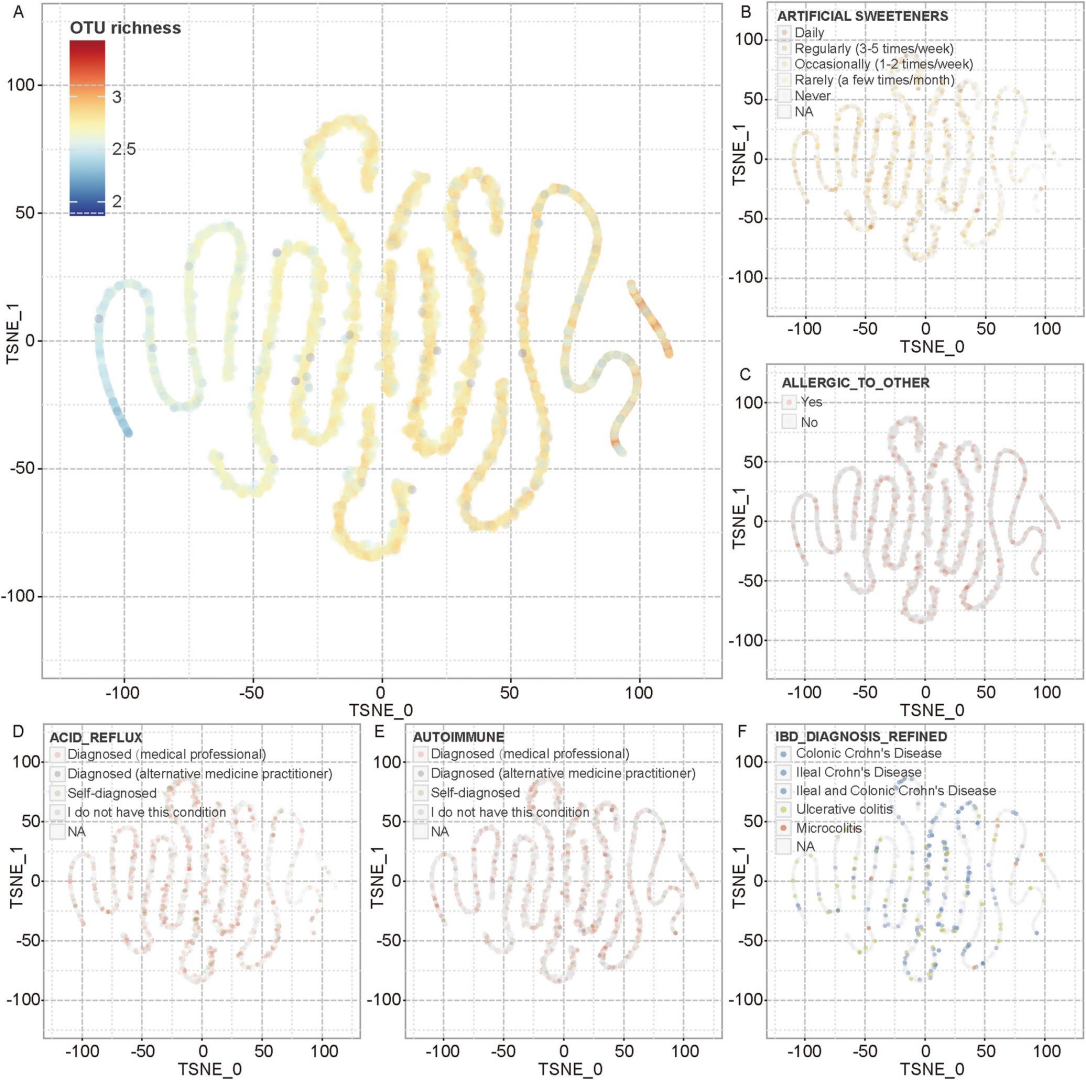
Samples in the sample space, colored by OTU richness (A) and sub‐attributes from five sample attributes (B–F). OTU, operational taxonomic unit.

The sample space provided another important result after the embedding of sample‐OTU networks. Figure 4 shows the distribution of 11,184 samples in the space. Unlike the distribution pattern of the OTU space, individuals in the sample space show a twisted‐rope‐like distribution (Figure 4A). In the sample space, the most evident factor related to sample distribution was the number of unique OTUs in each sample, which reflected the community richness of the sample’s alpha diversity. From the leftmost end of the “rope,” the richness contained in the sample tended to increase gradually. At both ends, samples tended to be more concentrated and the “rope” in these regions was thinner and more compact, yet samples in the middle were more dispersed and the rope were looser. Samples with higher richness tended to be distributed in the center of the rope, while samples with lower richness tended to be distributed around the rope.

To identify the relations between samples and their attributes, we labeled the sub‐attributes (referring to the different values of an attribute, see Section 3.1) of each phenotype or individual characteristics in the sample space. Figure 4B–F show some examples. It seems that no attribute clearly dominated sample distribution in the sample space. The global non‐dominance phenomenon of sample attributes could be found in all the 134 interested attributes in this study. The structure of the microbial community was influenced by a variety of factors, and the value of one attribute only constitutes a small part of all the influencing factors.

Despite the non‐dominance property from a global view, obvious local aggregations of some attributes could still be found if viewed locally. Figure S5 shows the distribution of different sub‐attributes of nine phenotypes or individual characteristics in a same region. There were many small clusters, which would be unlikely to form randomly, with several samples of the same sub‐attribute. To measure this local aggregation phenomenon, an average sample attribute neighbor distance (ASAND) was designed in this study. The ASAND value of each sub‐attribute was calculated and compared with the ASAND of random permutated samples, obtaining a *p*‐value as the LAI of the sub‐attribute (see Section 3.3.2). The LAI represents the spatial significance of the local aggregation of samples in a sub‐attribute. The lower the LAI is, the stronger the local aggregation is. More than 70% of all attributes observed in this study (96 out of 134) showed local aggregations (Table S2, LAI < 0.1). Sections 2.3.2–2.3.4 will further discuss the findings.

#### 2.3.2 Disease related phenotypes: Physical, nervous system, and allergy

Three types of sample attributes related to host‐disease were concerned in this study, including 21 phenotypes related to physical disease, 12 phenotypes related to nervous system disease, and 14 phenotypes related to allergy. Of those, phenotypes related to about 16 physical diseases, 12 nervous system diseases, and six kinds of allergies were detected as significantly locally aggregated (LAI < 0.1, Table S2). Most phenotypes that identified as locally aggregated have evidence in related literatures (Supporting Information S1: Supplementary Results). Specifically, phenotypes related to digestive tract disease tended to be more significant in LAI value. Among the 11 non‐digestive diseases, phenotypes related to cancer and cardiovascular diseases were most locally aggregated. All the nervous system related phenotypes and most food‐related phenotypes showed significant local aggregations; yet only one of the seven phenotypes related to non‐food allergies and seasonal allergies was detected as locally aggregated. The supporting literatures regarding the above results (Supporting Information S1: Supplementary Results) prove the validity of the methods of this study for extraction of medical semantic information and identification of the correlation between phenotypes and gut microbiomes. Although no phenotype information was used in the process of sample representation, the associations between gut microbes and phenotypes can be still effectively discovered by the LAI.

Meanwhile, this study also found that not all sub‐attributes of a phenotype were locally aggregated in the space. Among the significantly locally aggregated phenotypes found in this paper, the vast majority of locally aggregated sub‐attributes were of control samples (Supporting Information S1: Supplementary Results and Table S3), which may suggest that the gut microbial structure of healthy individuals has greater consistency than that of diseased individuals, which is the version of “all happy families are alike, but every unhappy family is unhappy in its own way” in the microbial world (Leo Tolstoy, Anna Karenina, 1878).

Despite the “Anna Karenina phenomenon,” some diseases still show consistent changes in the gut microbial structure. Table 2 presents disease sub‐attributes with significant local aggregations (LAI < 0.1). It can be seen that most locally aggregated disease sub‐attributes are associated with the gastrointestinal tract (IBD_DIAGNOSIS_REFINED, ACID_REFLUX), metabolic (DIABETES), and nervous system (VIVID_DREAMS, MENTAL_ILLNESS_TYPE_PTSD, MENTAL_ILLNESS_TYPE_BULIMIA_NERVOSA).

**TABLE 2:** Disease sub‐attributes with LAI < 0.1.

Type of phenotype	Phenotype	Sub‐attribute	LAI
Physical disease	IBD_DIAGNOSIS_REFINED	Colonic Crohn’s disease	0.0038
		Ileal and colonic Crohn’s disease	0.0771
	DIABETES	Diagnosed by a medical professional (doctor, physician assistant)	0.0417
	ACID_REFLUX	Self‐diagnosed	0.0737
	SKIN_CONDITION	Diagnosed by an alternative medicine practitioner	0.0552
Nervous system diseases	VIVID_DREAMS	Regularly (3–5 times/week)	0.0555
	MENTAL_ILLNESS_TYPE_PTSD^a^	Yes	0.0075
	MENTAL_ILLNESS_TYPE_BULIMIA_NERVOSA	Yes	0.0462
Allergy	GLUTEN	I was diagnosed with celiac disease	0.0034
	ALLERGIC_TO_TREE_NUTS	Yes	0.0816

Abbreviation: LAI, Local Aggregation Index.

^a^ MENTAL_ILLNESS_TYPE_PTSD is short for MENTAL_ILLNESS_TYPE_PTSD_POSTTRAUMATIC_STRESS_DISORDER.

There were phenomena linking with the disease sub‐attributes found in this study, that are worthy of further attention. One example is related to irritable bowel disorder, IBD. Among the five main IBD types, ulcerative colitis (UC), microscopic colitis (MC), and ileal Crohn’s disease (ileal CD) did not show significant local aggregations (Table S2), and only colonic Crohn’s disease (colonic CD) and ileal and colonic CD had LAI < 0.1 (the LAI of colonic CD was lower.) The difference between ileal and colonic CD in terms of the local aggregation might be related to the sampling technique of gut microbes. The ileum is part of the small intestine, the colon is part of the large intestine, and the AGP collects gut microbial samples by sequencing fecal samples. As UC, MC, and CD are all diseases of the colon, the difference in their local aggregation may be related to their different pathogenic mechanisms. The associations between vivid dreams and the gut microbiome are also interesting. Except the “Daily” sub‐attribute, the other four sub‐attributes of VIVID_DREAMS, from “Never,” and “Rarely” to “Occasionally” and “Regularly,” all showed significant aggregations (Table S2). It is hard to say whether having vivid dreams is a disease, but frequent vivid dreams exhaust people and may make people unable to distinguish dreams from reality, which is also related to diseases such as schizophrenia [84] and Parkinson’s disease [85]. There is a lack of research on the relationship between vivid dreams and gut microbiomes. The results of this study provide an inspiration that appropriate interventions in gut microbes may improve sleep quality by regulating the frequency of vivid dreams. Difference between phenotypes ALLERGIC_TO_TREE_NUTS and ALLERGIC_TO_PEANUTS is also thought‐provoking. In ALLERGIC_TO_TREE_NUTS, the sub‐attribute “Yes” was locally aggregated (LAI = 0.08), while the sub‐attribute “No” was not judged to be significant (LAI = 0.11). In ALLERGIC_TO_PEANUTS, only sub‐attribute “No” was locally aggregated (LAI = 0.07, and LAI of “Yes” is 0.90). Peanuts are often bracketed with tree nuts, and the results of this study suggested that their relationships with gut microbes may not be entirely consistent.

#### 2.3.3 Intake related individual characteristics: Diet, digest, and medication

The intake of food or medicine is another focus of this study. Unlike disease‐related phenotypes, the intake of food or medicine tends to be an active choice by individuals. Therefore, these characteristics may be more likely to be the cause, rather than the result, of the changes in the gut microbial structure. The results of the LAI analysis showed that most individual characteristics about dietary habits, medication or nutritional supplements, and digestive system health are also strongly related to the structure of the gut microbes.

More than 80% of dietary habit‐related individual characteristics (34 out of 41) showed significant local aggregations in at least one sub‐attribute, and some illuminating findings have been discovered (Supporting Information S1: Supplementary Results). Specific types of alcohol intake tend to have relatively consistent effects on the gut microbiome. On the contrary, the influence of specialized diet on the gut microbial structures seems more heterogeneous. In addition to the characteristics related to alcohol consumption and specialized diets, there were 24 characteristics of daily dietary habits, 20 of which showed significant aggregations (Table 3). Compared with disease‐related phenotypes, it is difficult to judge how a diet‐related individual characteristic can be “healthier.” However, most of their sub‐attributes with significant local aggregations found in this study are highly praised “healthy dietary habits” in some way. This may provide a “reference daily diet style” in view of this (Supporting Information S1: Supplementary Results).

**TABLE 3:** A list of “reference daily diet style” induced from LAI study on sample space.

Individual characteristic	Sub‐attribute	LAI
RED_MEAT_FREQUENCY	Rarely^a^	0.0456
MEAT_EGGS_FREQUENCY	Regularly^a^	0.0385
READY_TO_EAT_MEALS_FREQUENCY	Occasionally^a^, rarely	0.0902, 0.0838
WHOLE_EGGS	Occasionally (1–2 times/week)	0.001
MILK_SUBSTITUTE_FREQUENCY	Regularly, occasionally	0.0985, 0.0063
MILK_CHEESE_FREQUENCY	Daily	0.0003
TYPES_OF_PLANTS	11–20, 21–30	0.0002, 0.0062
VEGETABLE_FREQUENCY	Daily	0.0365
FERMENTED_PLANT_FREQUENCY	Rarely	0.0416
FROZEN_DESSERT_FREQUENCY	Rarely	0.0038
SUGARY_SWEETS_FREQUENCY	Rarely	0.0406
SUGAR_SWEETENED_DRINK_FREQUENCY	Never	0.0374
ARTIFICIAL_SWEETENERS	Never, rarely, regularly	0, 0.0001, 0.0002
ONE_LITER_OF_WATER_A_DAY_FREQUENCY	Occasionally	0.0622
SEAFOOD_FREQUENCY	Occasionally	0.057
OLIVE_OIL	Regularly, daily	0.0022, 0.0019
FRUIT_FREQUENCY	Occasionally	0.0023
WHOLE_GRAIN_FREQUENCY	Occasionally	0.043
PREPARED_MEALS_FREQUENCY	Occasionally	0.0505
HOMECOOKED_MEALS_FREQUENCY	Daily, regularly	0.0501, 0.0162

Abbreviation: LAI, Local Aggregation Index.

^a^ Rarely (less than once/week), occasionally (1–2 times/week), regularly (3–5 times/week).

This study focused on 10 characteristics related to medication or nutritional supplements, among which seven characteristics showed significant local aggregations (Table S2). Specifically, people who do not consume vitamin B or vitamin D had stronger local aggregations in the sample space. While for multivitamins, no significant local aggregation was found, regardless of whether individuals were taking them or not. This phenomenon implies that single vitamin supplements may have a greater impact on the gut microbes. Individuals who had received the flu vaccine showed significant local aggregations (FLU_VACCINE_DATE, “Week” and “6 months”). Although the impact of the flu vaccine on the gut microbiome structure of human bodies has not been sufficiently studied, it has been reported that the gut microbiome may alter the body’s immune response to the flu vaccine [86], and different types of viruses change the gut microbes in different ways [87]. Two characteristics, ACNE_MEDICATION and ACNE_MEDICATION_OTC, did not show any aggregation, which is reasonable because acne medications are largely topical and applied to a small area.

Diet and medication intake affect intestinal digestion, absorption function, and metabolism. Specifically, according to the calculation of the LAI of four sample attributes of the digestive system (Table S2), stable body mass index, BMI, did not cause significant local aggregation of gut microbiomes. However, individuals who gained 10 pounds in a short period of time showed significant local aggregations, suggesting that rapid weight gain to obesity may be related to specific mechanisms of microbial communities in vivo.

#### 2.3.4 Other characteristics or features that may impact gut microbiome

This study focused on six individual demographic characteristics, including SEX, AGE_CAT, LEVEL_OF_EDUCATION, ECONOMIC_REGION, RACE, and CENSUS_REGION, five among which did not have any sub‐attribute that showed significant local aggregation. The only exception was “Graduate or Professional degree” in LEVEL_OF_EDUCATION, which is the highest degree among the seven options. Studies have shown that better educated people tend to consume healthier diets [88]. This may explain the results found in this study to some extent. The least local aggregation tendency was found in RACE, with the LAI values of all its five sub‐attributes >0.3, which illustrates racial equality from the perspective of our microbial partners.

Among the 19 individual characteristics regarding life and habits, eight showed significant local aggregations, including DRINKING_WATER_SOURCE (“City”), LAST_MOVE (“I have lived in my current state of residence for more than a year.”), ROOMMATES (“More than three”), EXERCISE_LOCATION (“Indoors”), PETS_OTHER (“No”), EXERCISE_FREQUENCY (“Occasionally [1–2 times/week]”), FLOSSING_FREQUENCY (“Rarely [a few times/month]”), and CONSUME_ANIMAL_PRODUCTS_ABX (“Yes”). Most of these sub‐attributes depict an image of “a group with consistent intestinal flora,” with features such as living in cities, not moving frequently, living with more people, going to the fitness center once or twice a week, using dental floss occasionally, not rejecting animal products, etc.

Three pregnancy and infants related characteristics were studied, including CSECTION, FED_AS_INFANT, and PREGNANT. Significant local aggregations were seen in individuals who were born by vaginal delivery and were predominantly breastfed during infancy. Although PREGNANT was not judged to be significantly aggregated, the LAI of its sub‐attribute “No” was 0.11, indicating that pregnancy is also associated with the gut microbial structure to some extent.

In addition, another noteworthy phenomenon was that factors related to the experiments could also affect the local aggregation of the samples. This study found that both COLLECTION_MONTH and COLLECTION_SEASON had significantly locally aggregated sub‐attributes. In COLLECTION_SEASON, the LAI of Winter was 0. In COLLECTION_MONTH, the LAI values of January, February, and March were the lowest, which were 0.0001, 0.0006, and 0.0813, respectively, but the LAI values of November and December were not low (0.47 and 0.44, respectively), suggesting that the reason for the significant aggregation of samples may not be the season and temperature, but a batch effect.

### 2.4 Classification effect based on OTUs vectors

#### 2.4.1 Pre‐trained OTU vectors help deep learning model to perform better

In addition to the perspective of analyzing local aggregations using LAI, there is another approach to understanding samples, which is to classify the samples and identify relevant features that contribute to the classification. This study developed an IMETA (Section 3.4.1). The model integrates the functional information contained in OTU vectors with the original abundance information, and attempts to understand the biological characteristics of the OTUs that contribute to classification in terms of the attributes of the samples.

In order to demonstrate the value of using the pre‐trained OTU vectors that are proposed in this paper, an end‐to‐end neural network model is developed and compared with the IMETA model. The end‐to‐end model shares a similar overall structure to that of IMETA, but it does not rely on any pre‐trained OTU embedding vectors. Instead, it directly learns low‐dimensional representations of OTUs, the importance of OTUs, and attribute prediction parameters, from the prediction task.

To verify the classification performance of the IMETA model and to explore the results of the classification tasks for different phenotypes or individual characteristics, this study selected 26 attributes of interest with more than 100 positive and negative samples. Among the 26 attributes, some showed significant local aggregations in the sample space according to the LAI, while others did not. This study regarded the classification of each attribute as a separate task. In each task, sub‐attributes were first binarized into 0/1, and IMETA was then employed to perform the classification (Tables S4 and S5). To avoid randomness during the deep learning model training process, each model was run five times, and their average performance was then considered. The results (Table S4) showed that the IMETA model outperformed the end‐to‐end model in more than two‐thirds (18 out of 26) of the tasks. Additionally, among the five tasks that had at least one *F*1 score above 0.7 (indicating a clearer pattern of attribute classification), the IMETA model consistently outperformed the end‐to‐end model in terms of classification accuracy.

#### 2.4.2 Superior performance of IMETA compared to classic models

In addition to comparing IMETA with end‐to‐end models that do not use pre‐trained vectors, this study also compared it with some classic interpretable classification models as baseline models, including random forest (RF), linear support vector machine, and logistic regression (LR). Among the 26 attribute classification tasks used for comparison, 21 tasks had sample sizes of over 5000, which can be considered as sufficient sample sizes, while the sample sizes of the other 5 tasks were all <3500, and these were considered as small sample sizes. This study evaluated the performance of IMETA and various baseline methods in both tasks with sufficient sample sizes and tasks with small sample sizes in terms of *F*1 score.

The overall trends of the performance of each method on the different classification tasks are relatively consistent, reflecting the difficulty of the classification tasks. Tables S4 and S5, respectively, illustrates the performance of each method in each attribute classification task for the two sample size groups (S4 for large sample size and S5 for small sample size). Among the attributes in the large sample size group, the following obtained the best classification results: IBD_DIAGNOSIS_REFINED, ALCOHOL_FREQUENCY, COLLECTION_SEASON, LAST_TRAVEL, and VITAMIN_D_SUPPLEMENT_FREQUENCY. The IMETA got an *F*1 score >0.6 in the classification tasks for all five attributes (Table 4). The classification task for the attribute IBD_DIAGNOSIS_REFINED was to distinguish individuals and controls with colonic CD in the samples. All five models provided satisfactory classification results, indicating that the gut microbiota features of colonic CD patients are distinct. In the classification tasks of the other four attributes, the IMETA achieved the best or very close to the best performance, indicating that the IMETA has an excellent and stable classification performance for problems with certain separability. Among these five attributes, LR had the second‐best performance after the IMETA. However, compared with LR, the IMETA showed a greater advantage in terms of speed; taking the LAST_TRAVEL classification task with about 9000 training samples as an example, LR required more than 40 min for training and prediction, while the IMETA only needed 5 min.

**TABLE 4:** Performances of the five large sample size classification tasks with highest *F*1 scores.

Classification task (represented by attribute name)	IMETA	End‐to‐end^a^	Linear SVM^b^	Random forest^b^	Logistic regression^b^
IBD_DIAGNOSIS_REFINED	**0.9565**	**0.9565**	**0.9565**	**0.9565**	**0.9565**
ALCOHOL_FREQUENCY	**0.8104**	0.7873	0.7700	0.7024	0.7923
COLLECTION_SEASON	0.7288	0.7215	0.7014	0.6869	**0.7295**
LAST_TRAVEL	**0.7133**	0.6942	0.6171	0.6458	0.6532
VITAMIN_D_SUPPLEMENT_FREQUENCY	**0.6098**	0.5842	0.5246	0.5743	0.5946

*Note*: The bold values indicate the optimal performance in each task.

Abbreviations: IMETA, Integrated Model of Embedded Taxonomies and Abundance; OTU, operational taxonomic unit; SVM, support vector machine.

^a^ The end‐to‐end model refers to a model with the same structure as IMETA, but without any pre‐training of OTU representation vectors, and only relying on attribute classification tasks for end‐to‐end training.

^b^ The implementation and parameters of linear SVM, random forest, and logistic regression can be found in Section 3.4.2.

The model structure of the IMETA enables its interpretability, that is, it has the ability to explain the importance of features for classification. In the 1D convolution layer of the IMETA model, there are three convolution kernels, each of which is equivalent to a weighted sum of the representation vectors of various OTUs. The larger the absolute value of the parameter corresponding to a certain OTU, the higher the importance of that OTU. To show the interpretability of the IMETA, parameters of the 1D convolution layer of the trained model were extracted using the attribute IBD_DIAGNOSIS_REFINED as an example. The parameter values of the three convolution kernels were averaged to generate an “importance score” and analyzed in the vector space to gain insight.

Figure S6 displays the spatial distribution of OTUs that are considered most valuable for the classification of the IBD_DIAGNOSIS_REFINED attribute in IMETA. Out of all 23,390 OTUs used for classification, only 31 OTUs had an importance score >0.3. These OTUs were not distributed in the regions with the highest prevalence. Instead, the highest prevalence among them was only 1951, meaning that the “important OTUs” for classification were distributed in <18% of all 11,089 samples. Some OTUs with relatively high importance scores were noticeably clustered together in local regions. Figure S7 provides an example of a local area, where microbes, including Dorea [89], Methylophilaceae [90], Ruminococcaceae [91], Fusobacterium [92], and Clostridium [92], have been reported to be significantly associated with colonic cancer or adenoma, suggesting a consistent role of these microbes in colonic health within this region. Compared to traditional classification tools that can also report the importance of OTUs in classification, the understanding of the functionality of these OTUs can only be inferred based on individual OTU classification annotations, making it difficult to obtain a more accurate understanding. In contrast, the method used in this study can cluster OTUs with similar functions together, and combined with OTU classification annotations, it can provide somewhat more precise information on the explicability of classification problems.

## 3 MATERIALS AND METHODS

### 3.1 Data acquisition and preprocessing

In this study, the latest version of the OTU table and metadata of gut microbes were downloaded from the FTP server of AGP. The original OTU table included 15,158 samples and 36,405 unique OTUs. To obtain reliable abundance information on OTUs and make the results of subsequent analyses, such as network embedding, more accurate, this study screened the sequencing depth of the samples, and only the samples containing more than 10,000 OTUs, and OTUs that at least appeared in more than one sample, were retained. After the screening, 11,184 samples and 23,464 OTUs were retained.

The metadata of AGP dataset contains attributes of each sample, facilitating researchers to study the associations between microbiota and these attributes. In this study, 134 attributes of interest were selected from the metadata provided by AGP and divided into 10 categories (Table 5). Categorized into three types, one type is a “phenotype,” such as physical diseases, nervous system diseases, allergies, and situations related to the digestive system. Another type is “sample characteristic,” including demographic characteristics, lifestyle and customs, and medication or nutritional supplements. The third type is a “sample feature,” such as collection month or season. They are collectively referred to as “sample attributes.” In the metadata, the AGP team used uppercase letters to name these sample attributes, for example, “IBD_DIAGNOSIS_REFINED.” This paper adopts this expression style.

**TABLE 5:** Attributes investigated in this research.

Category	Number of attributes	Examples^a^
Demographic characteristics	6	SEX, AGE (category), LEVEL_OF_EDUCATION, ECONOMIC_REGION, RACE, CENSUS_REGION
Life and habits	21	DRINKING_WATER_SOURCE, LAST_MOVE, ROOMMATES, EXERCISE_LOCATION, LAST_TRAVEL, CAT, DOG, PETS_OTHER, SLEEP_DURATION, EXERCISE_FREQUENCY, POOL_FREQUENCY, …
Dietary habits	41	ALCOHOL_FREQUENCY, DRINKS_PER_SESSION, ALCOHOL_TYPES related characteristics (six attributes), SPECIALIZED_DIET related characteristics (nine attributes), RED_MEAT_FREQUENCY, MEAT_EGGS_FREQUENCY
		ARTIFICIAL_SWEETENERS, OLIVE_OIL, FRUIT_FREQUENCY, …
Medication or nutritional supplements	10	ACNE_MEDICATION, CANCER_TREATMENT, FLU_VACCINE_DATE, ANTIBIOTIC_HISTORY, MULTIVITAMIN, PROBIOTIC_FREQUENCY, …
Physical disease	21	IBD, IBS, SIBO, DIABETES, MIGRAINE, CARDIOVASCULAR_DISEASE, LUNG_DISEASE, LIVER_DISEASE, KIDNEY_DISEASE, THYROID, …
Nervous system diseases	12	VIVID_DREAMS, EPILEPSY_OR_SEIZURE_DISORDER, MENTAL_ILLNESS_TYPES related phenotypes (seven attributes), ADD_ADHD, ASD, ALZHEIMERS, …
Digestive system	4	BMI (category), WEIGHT_CHANGE, BOWEL_MOVEMENT_QUALITY, BOWEL_MOVEMENT_FREQUENCY
Allergy	14	GLUTEN, SEASONAL_ALLERGIES, FOOD_ALLERGIES related phenotypes (six attributes), NON_FOOD_ALLERGIES related phenotypes (six attributes)
Pregnancies and infants	3	CSECTION, FED_AS_INFANT, BREASTMILK_FORMULA_ENSURE, PREGNANT
Other	2	COLLECTION_MONTH, COLLECTION_SEASON

^a^ Examples show representative attributes of corresponding categories in this study.

All samples attributes have specific values. For example, phenotype “IBD_DIAGNOSIS_REFINED” can take one of the following six values: colonic CD, Ileal and colonic CD, ileal CD, UC, Microcolitis, and NA. These values are all collectively referred to as “sub‐attributes” in the paper. Due to the extended duration of the AGP project spanning several years and various factors during the collection of samples in different batches (e.g., design of volunteer survey questionnaires, recording by sampling staff), the same attribute values may be expressed differently in the metadata of samples from different batches. Consequently, different values of attributes in the metadata may carry the same meaning, such as “Yes”/“No” and “True”/“False.” These different expressions will be treated as distinct values of attributes by the computer. Therefore, this study consolidated and combined the values of these synonyms. For example, sub‐attributes “Unspecified,” “unspecified,” “Unknown,” “Not sure,” “no_data,” “nan,” “Missing: Not provided,” “Not provided” were standardized into “NA,” and sub‐attributes “true” and “false” were changed into “Yes” and “No,” respectively.

### 3.2 Network construction and embedding

The OTU table provided by AGP contains the number of read counts of each OTU in each sample. To avoid the influence of the difference in sequencing depth of different samples on the analysis results, the read counts was converted into relative abundances of OTUs in the samples. Network embedding relies on meta‐path random sampling, and in this study, the selection of the next node in random sampling was correlated with the weight of the edge connected to the node. To avoid excessive concentration of sampling on the dominant OTUs of each sample, due to the huge difference between the abundance of dominant OTUs and rare OTUs, this study focused on the variation of OTU abundance between samples (for example, the difference between the abundance of OTU 4,385,479 and that of OTU 4,424,063 exceeded that in sample 10,317.000065535 by a factor of 10^4^). Therefore, in the sample‐OTU weighted heterogeneous information network constructed based on the OTU table, relative abundance values were first log‐transformed and standardized, then normalized to between 1 and 7 as the edge weights. The equation is given as follows:

$$w_{ij}=\left\{\begin{array}{cc} 7, & \text{if}\log_{10}r_{ij}-\mu_{j}\geq 3\sigma_{j}\\ \frac{\log_{10}r_{ij}-\mu_{j}}{\sigma_{j}}+4, & \text{if}-3\sigma_{j}<\log_{10}r_{ij}-\mu_{j}<3\sigma_{j,}\\ 1, & \text{if}\log_{10}r_{ij}-\mu_{j}\leq -3\sigma_{j}\\ 0, & \text{if}r_{ij}=0 \end{array}\right.$$

where *w*_*ij*_ represents the weight of the edge between the *i*th sample and the *j*th OTU; *r*_*ij*_ represents the relative abundance of the *j*th OTU on the *i*th sample; $\mu_{j}=\left(1/n_{j}\right)\sum_{i=1}^{n_{j}}\;\log_{10}r_{ij}$ and $\sigma_{j}={\left(\left(1/n_{j}\right)\sum_{i=1}^{n_{j}}{\left(\log_{10}r_{ij}-\mu_{j}\right)}^{2}\right)}^{\frac{1}{2}}$ represent the mean and standard deviation values, respectively, of the log_10_ abundance of the *j*th OTU of the all *n*_*j*_ samples where the abundance of the OTU is not zero. The network constructed directly reflected the interactive relationship between the OTUs and the samples, which included two types of nodes, namely OTU nodes and sample nodes. The weights of the edges between the two types of nodes reflected the tendency of an OTU to increase or decrease in a sample compared to the overall population of all samples in the dataset.

MetaPath2Vec was used to embed and represent nodes as low‐dimensional vectors in the above network, and a random walk sampling of the heterogeneous information network was conducted based on metapath OTU‐Sample‐OTU (O‐S‐O). A heterogeneous skip‐gram model was used to analyze the sampled sequences, and embedding representation was performed for each type of node. In this study, the random walk approach was modified for the weighted heterogeneous network, with the transition probability of each step given by the following equation:

$$p\left(v^{t+1}|v^{t}\right)=\frac{w_{v^{t}v^{t+1}}}{\sum_{v\in Nb\left(v^{t}\right)}w_{v^{t}v}},$$

where *v* ^*t*^ and *v* ^*t*+1^ denote the nodes sampled by the walker at the *t*th step and the (*t* + 1)th step, respectively; *Nb*(*v* ^*t*^) represents the set composed of all neighbors of node *v* ^*t*^; $w_{v^{t}v}$ is the weight of the edge between node *v* ^*t*^ and node *v* in the network. In short, in the random walk, the random selection of the next node *v* ^*t*+1^ should be based on the proportions of the weights of all the edges of the current node *v* ^*t*^. In the network of this study, as edges only exist between the OTUs and the samples, the next node of each sample node must be an OTU node, and each OTU node must be followed by a sample node. This study started from each OTU and sampled the network 100 times, and the length of each sampling was 100 O‐S‐O cycles (i.e., walking from an OTU to a sample and then from a sample to an OTU). The parameter of MetaPath2Vec was “‐pp 1 ‐size 128 ‐window 7—negative 5,” and 128‐dimensional OTU vectors and sample vectors were obtained.

### 3.3 Basic concepts and statistics used in this study

#### 3.3.1 Prevalence and average abundance of OTUs

The prevalence of an OTU refers to the number of occurrences of the OTU in all samples. The average abundance of an OTU in this paper is specifically defined as its relative abundance among the samples where it occurs, and not across the entire dataset. Some OTUs have a very low prevalence, but their abundance is not low in single samples. Other OTUs have a relatively higher prevalence, but their abundance is very low in single samples. This approach can avoid the problem that the calculation of average abundance based on all samples is excessively affected by prevalence. The average abundance $r_{j}^{o}$ is calculated as follows:

$$r_{j}^{o}=\frac{1}{n}\sum_{i=1}^{n_{j}}r_{ij},$$

where *r*_*ij*_ represents the relative abundance of the *j*th OTU on the *i*th sample; *n*_*j*_ represent the number of samples on which the abundance of the *j*th OTU is not zero.

#### 3.3.2 Local Aggregation Index

First, for each sub‐attribute *p*_sub_, an ASAND was calculated, as shown below:

$$\text{SAN}D_{s_{i}}=\min_{s\in D_{s_{i}},s\neq s_{i}}\rho_{ss_{i}},$$

$$\text{ASAN}D_{p_{\text{sub}}}=\frac{1}{N_{p_{\text{sub}}}}\sum_{s_{i}\in D_{p_{\text{sub}}}^{\text{top}10\%}}\text{SAN}D_{s_{i}},$$

where $D_{s_{i}}$ denotes the set of samples with the same sub‐attribute as *s*_*i*_; $\rho_{ss_{i}}$ represents the Euclidean distance between sample *s* and sample *s*_*i*_; $\text{SAN}D_{s_{i}}$ means taking the minimum (i.e., calculating the distance between *s*_*i*_ and its closest neighbor with the sub‐attribute). The local aggregation in this study aims to measure the tendency of samples to be aggregated. To make this tendency more obvious, for each sub‐attribute, ASAND in this study only took into account the most aggregated part of the sample set, namely the top 10% samples with the shortest SANDs. The set of these samples is $D_{p_{\text{sub}}}^{\text{top}10\%}$, and the sample size is $N_{p_{\text{sub}}}$.

The LAI was established based on the ASAND. The ASAND values of specific sub‐attributes were compared with the ASAND values after a random permutation to determine whether the sub‐attribute caused significant local aggregation of the sample:

$$I\left(\text{ASAN}D_{p_{\text{sub}}}^{\text{rando}m_{i}}\right)=\left\{\begin{array}{cc} 1, & \text{if}\;\text{ASAN}D_{p_{\text{sub}}}^{\text{rando}m_{i}}\leq \text{ASAN}D_{p_{\text{sub}}}^{\text{real}}\\ 0, & \text{if}\;\text{ASAN}D_{p_{\text{sub}}}^{\text{rando}m_{i}}>\text{ASAN}D_{p_{\text{sub}}}^{\text{real}} \end{array}\right.,$$

$$\text{LAI}=\frac{\sum_{i=1}^{10,000}I\left(\text{ASAN}D_{p_{\text{sub}}}^{\text{rando}m_{i}}\right)}{10,000},$$

where $I\left(\text{ASAN}D_{p_{\text{sub}}}^{\text{rando}m_{i}}\right)$ is an indicative function. For the *i*th permutation, when, and only when, the calculated $\text{ASAN}D_{p_{\text{sub}}}^{\text{rando}m_{i}}$ is lower than the $\text{ASAN}D_{p_{\text{sub}}}^{\text{real}}$ based on the real sample label, it takes a value of 1. The LAI counts all experiments in the permutation that are more aggregated than the real situation and calculated the proportions. A smaller value indicates a more significant local aggregation of the sub‐attribute.

### 3.4 Model and experimental design

#### 3.4.1 Proposed model in this study

**FIGURE 5:**
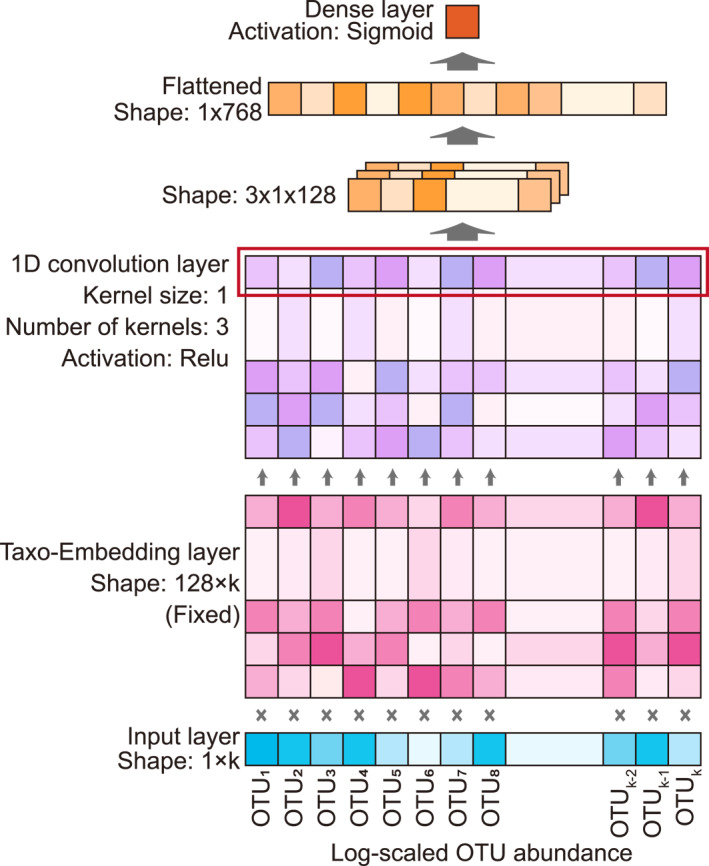
Structure of Integrated Model of Embedded Taxonomies and Abundance model.

For the sample classification task based on attributes, this study proposed an IMETA, a deep learning model that can make full use of the original OTU abundance information together with the biological characteristic information of OTUs and samples extracted from the sample–OTU interactions to predict whether a sample belongs to an attribute class. Figure 5 shows the network structure of the IMETA. The input of the model includes two parts: a base 10 log‐scaled OTU abundance vector and a 128‐dimensional sample vector embedded through the network. The OTU abundance vector has k dimensions, with each dimension corresponding to an OTU that is present at least once in either the training or test set. Taxo‐Embedding was performed on each OTU (i.e., multiplying the log‐scaled relative abundance of each OTU in the sample by the 128‐dimensional embedding vector of the OTU embedded through the sample‐OTU network) to embed the k‐dimensional OTU abundance vector of the sample as a 128 × k dimensional matrix. This matrix went through the 1D convolution layer with three kernels to form three 1 × 128 dimensional vectors. The computation in each convolution kernel is equivalent to the weighted sum of embedded OTU vectors, which includes abundance information. The 1D convolution kernel encapsulates the importance of each OTU for classification. The three 128‐dimensional vectors were then flattened to form a 768‐dimensional vector, and after a dense layer the binary classification result of whether the sample belongs to an attribute class was output. Since for most attributes, positive and negative samples are unbalanced, with negative samples accounting for a larger proportion. Some attributes even have more than 99% negative samples. To avoid the classifier’s tendency to classify samples as negative ones to obtain better accuracy, this study performed up‐sampling of the category with fewer samples in each classification task to ensure a balance between the two types of samples.

The computation in each convolution kernel is equivalent to the weighted sum of each embedded OTU vector, incorporating abundance information. The 1D convolution kernel encapsulates the importance of each OTU for classification.

#### 3.4.2 Compared methods and evaluation

The baseline methods, basic parameters, and necessary data preprocessing methods used in this paper are shown in Table 6. In this paper, the IMETA model is compared with four baseline methods, one of which is an end‐to‐end model without any pre‐training of OTU representation vectors. IMETA utilizes a set of pre‐trained OTU vectors that are achieved through the construction and embedding host–microbe heterogeneous networks. The comparison with the end‐to‐end model aims to showcase the advantages of employing pre‐trained OTU vectors in comprehending the underlying functional mechanisms of host and microbes. The other three compared methods are classic interpretable classification models, used to test the characteristics of the IMETA in explaining the importance of OTUs for attribute classification. All the parameters of the three classic models were selected by grid search to find the best‐fitting combinations. During the parameter optimization process, considering the computational complexity, five representative attributes were initially selected, and the model parameters were chosen based on the overall performance of the models on these five attribute classification tasks. The selected parameters are shown in Table 6.

**TABLE 6:** Parameters of the classic interpretable classification models.

Compared method^a^	Parameters^b^	Data preprocessing
Linear SVM	*C* = 1, class_weight = “balanced,” kernel = “linear”	Log‐scaled
Logistic regression	*C* = 0.1, class_weight = “balanced,” penalty = “l1”	Log‐scaled
Random forest	class_weight = “balanced,” max_depth = 5, n_estimators = 100	Log‐scaled

^a^ The three compared methods were all implemented using the sklearn package in Python (SVC, RandomForestClassifier, and LogisticRegression).

^b^ The term “Parameters” refers to the parameters of the corresponding sklearn model.

This study mainly used *F*1 scores to evaluate the performance of various methods in classification tasks. Besides, *specificity, recall, precision, false discovery rate,* and *accuracy* are also used and their calculation methods are shown in Table 7. *True positive* indicates the number of samples correctly predicted as the positive class, while *true negative* represents the number of samples correctly predicted as the negative class. *False positive* represents the number of samples that are predicted as positive but are actually negative, and *false negative* represents the number of samples that are predicted as negative but are actually positive.

**TABLE 7:** Metrics and their calculation.

Metrics	Calculation
*Specificity*	$\frac{TN}{TN+FP}$
*Recall*	$\frac{TP}{TP+FN}$
*Precision*	$\frac{TP}{TP+FP}$
*FDR*	$\frac{FP}{TP+FP}=1-Precision$
*Accuracy*	$\frac{TP+TN}{TP+FP+TN+FN}$
*F*1 score	$2\times \frac{Precision\times Recall}{Precision+Recall}$

Abbreviations: *FDR*, *false discovery rate*; *FN*, false negative; *FP*, false positive; *TN*, true negative; *TP*, true positive.

## 4 DISCUSSION

This study used the heterogeneous network embedding method to reconstruct the host–microbe interaction information contained in the OTU table into a low‐dimensional vector that reflects the biological characteristics of OTUs and samples. Results demonstrate that this is a reasonable biological semantic representation. The IMETA that uses this information can achieve better performance in attribute classification tasks compared with an end‐to‐end model, and it is faster, more accurate, and more interpretable for large sample sizes than traditional interpretable classification models. The superiority of the IMETA further verifies the validity of the embedded vectors in terms of understanding function OTUs and samples.

End‐to‐end models have strong capabilities in both embedding and prediction. There may be two reasons why the IMETA outperforms the end‐to‐end model. In the IMETA, although the training of pre‐trained Taxo‐Embedding layer is irrelevant to the target attribute, it contains information from all samples for OTU functional representation because its parameters come from the embedding matrix of the whole host–microbe heterogeneous network. In this view, its information perspective is different from that obtained during classification learning, so the information in the pre‐training vectors and the training data can complement each other to some extent. At the same time, in the IMETA, since the Taxo‐Embedding layer is fixed, it requires relatively fewer parameters to be trained, and its generalization ability tends to be stronger in the case of a limited sample size.

This study established the LAI to measure whether attributes show local aggregations in the sample space. A smaller LAI value possibly suggests a greater impact of gut microbes on the attribute. However, this simple measurement still has much room for improvement. On the one hand, due to the complexity of the living habits and health of human beings, the attributes discussed in this study, such as phenotypes or individual characteristics, are not completely independent from each other. The LAI value of a single sub‐attribute may not fully reflect the relationship between the attribute and the microbiome; it could be associated with other attributes that exhibit a strong correlation with the microbiome structure. On the other hand, an insignificant LAI value does not necessarily mean that the attribute does not affect the gut microbes. It can also suggest that the attribute does not affect the gut microbial structure of different individuals in the same direction. Therefore, the significance of the values of LAI can only be used as a reference for exploring the association between the host attribute and the gut microbes.

This study focuses on the potential help of network embedding for understanding the properties of OTUs and the meanings of attributes. Some methods, such as the network embedding procedure used in this paper and the proposed IMETA model, are simple and worth further exploration. For example, compared with the sample‐OTU bipartite graph, more complex network structures can be built. The IMETA model can also be improved; for example, the parameters of the Taxo‐Embedding layer can be trained task‐by‐task using transfer learning approach for attribute classification. This approach not only overcomes the problem of relatively small sample size and inability to include all sample information in single‐task end‐to‐end learning but also provides a better understanding of microbial function from multiple attribute perspectives. In addition, limited by the sample size, adding some regularization to the model may further improve its performance. Although the IMETA has the advantages of faster running speed, higher accuracy, and stronger interpretability when the sample size is sufficient and the attribute is highly distinguishable, it should be acknowledged that its classification performance is not superior to traditional classification methods when the sample size is small. Therefore, developing an IMETA deep learning model suitable for small sample sets is also a valuable direction for future research.

It is hoped that this paper can inspire an understanding of microbial functional characteristics, host phenotypic and individual characteristics, and the underlying mechanisms of the host–microbe relationship.

## AUTHOR CONTRIBUTIONS

**Hongfei Cui**: Conceptualization; data curation; methodology; validation; visualization; writing; funding acquisition.

## CONFLICT OF INTEREST STATEMENT

The author Hongfei Cui declares that she has no conflict of interest or financial conflicts to disclose.

## ETHICS STATEMENT

All procedures performed in studies involving animals were in accordance with the ethical standards of the institution or practice at which the studies were conducted, and with the 1964 Helsinki declaration and its later amendments or comparable ethical standards.

## Supporting information

Supporting Information S1

Table S1–S5

## Data Availability

The host–microbe interaction information and host metadata can be obtained from the OTU table of fecal samples in the American Gut Project.
