# Supplementary material for: Reorganizing heterogeneous information from host–microbe interaction reveals innate associations among samples
Source: Quant Biol. 2023 Nov 29;11(4):451–70. doi: 10.1002/qub2.25 (PMC12807269; doi:10.1002/qub2.25)
Supplement: Supplementary file 1 — Supporting Information S1 [file QUB2-11-451-s002.pdf]

Supplementary Results

1. Taxonomy distribution patterns in OTU space

As taxonomy is also related to the biological functions of OTUs to some extent, the distribution of taxonomy in OTU space was investigated in this study. First, distributions of OTUs were observed on phylum level. For each phylum, although OTUs tend to be generally distributed throughout the space, different phylum still exhibited very distinctive distribution patterns in terms of the density of OTUs. Take the five phyla containing the largest numbers of OTUs as examples (Supplementary Fig. S1), most Firmicutes were clustered in the left croissant, except for the three small clusters next to it (Supplementary Fig. S1A, green box). In contrast, Proteobacteria were more distributed in the right croissant, and OTUs belonging to Proteobacteria in the left croissant were concentrated on the inner side (Supplementary Fig. S1B, red dashed line) and in the upper-left and right-hand clusters of the three small clusters (Supplementary Fig. S1B, red boxes). Bacteroidetes were relatively evenly distributed in the whole space (Supplementary Fig. S1C), and the clustering of Actinobacteria was evident around the three clusters next to the left croissant (Supplementary Fig. S1D). Acidobacteria were mainly distributed in the low abundance region. In addition to the right croissant (Supplementary Fig. S1E, red dashed line), and they were also distributed on the outer side of the lower left part of the left croissant and the cluster closest to the left croissant (Supplementary Fig. S1E, red box). Moreover, there was an interesting phenomenon that there was an obvious cluster in the fourth quadrant (Supplementary Fig. S1F, from the blue boxed region of Supplementary Fig. S1A-E) in addition to the regions of the two croissants. In this cluster, only two Acidobacteria OTUs and two Firmicutes OTUs could be found. Most OTUs distributed in this region were Proteobacteria, Bacteroidetes, and Actinobacteria. Other phyla had their respective characteristics of distribution. The OTUs of some phyla tended to be distributed in the right croissant, such as Planctomycetes (Supplementary Fig. S2A) and Chloroflexi (Supplementary Fig. S2B), while the OTUs of some other phyla tended to be distributed in the left croissant, such as Tenericutes (Supplementary Fig. S2C) and Fusobacteria (Supplementary Fig. S2D).

The distribution of OTUs was highly dispersed even at the species level. Supplementary Fig. S3A-C shows the distribution of the OTUs of species *Faecalibacterium prausnitzii* (*F. prausnitzii*, Supplementary Fig. S3A), a core gut microbe, and the distribution of the OTUs of the family (Ruminococcaceae, Supplementary Fig. S3B) and the class (Clostridia, Supplementary Fig. S3C) to which *F. prausnitzii* belongs to. Supplementary Fig. S3D-F shows the distributions of OTUs from Faecalibacterium's three main genera (*Faecalibacterium*, *Oscillospira*, and *Ruminococcus*). It could be seen that OTUs with lower taxonomy levels showed more heterogeneity in the spatial distribution. However, OTUs at the same species level would be widely distributed across the space. For microorganisms, genetic stratifications and phenotypic classification may differ greatly. A slight difference in the living environment or genotypic differences can result in huge changes in a microbe's phenotype character[1]. The findings of the present study are consistent with this understanding.

2. Disease related local aggregated phenotypes and their sub-attributes

Supplementary Table S3 illustrates phenotypes with significant local aggregation in the categories of physical disease or nervous system disease with LAI < 0.01, along with the sub-attributes in which local aggregations occurred. It can be seen that among the 21 physical disease phenotypes, nine phenotypes have sub-attributes that show significant (LAI<0.01) local aggregations, among which there are seven digestive tract disease phenotypes, including five intestinal disease phenotypes (IBD_DIAGNOSIS_REFINED、IBS、SIBO、FUNGAL_OVERGROWTH、CDIFF), one gastric disease phenotype (ACID_REFLUX), and one liver disease phenotype (LIVER_DISEASE). CARDIOVASCULAR_DISEASE and CANCER, two diseases not directly related to the digestive tract, also showed very significant aggregation in the sample space of gut microbes. In addition, seven physical disease phenotypes (EBD, DIABETES, MIGRAINE, KIDNEY_DISEASE, THYROID, AUTOIMMUNE, and SKIN_CONDITION) showed significant local aggregations (LAI<0.1, Supplementary Table S2). The remaining five physical disease phenotypes without significant local aggregation are DIABETES_TYPE, LUNG_DISEASE, TONSILS_REMOVED, CHICKENPOX, and PKU (Phenylketonuria). Above results indicate that the structure of gut microbes more related to digestive tract diseases, and the association between some non-digestive tract diseases and the structure of gut microbes may be related to a variety of factors such as intestinal metabolites and immune regulation. Some studies have demonstrated that some metabolites of gut microbes can affect human health. For example, trimethylamine-N-oxide (TMAO) is associated with an increased risk of cardiovascular diseases such as atherosclerosis and thrombosis[2,3] and is significantly increasing in patients with chronic nephritis[4–6]. Gut microbial dysbiosis can destroy the mucosal layer so that antigens can more easily pass through the intestinal barrier, triggering an increase in inflammatory factors in the body and thus leading to the development of diseases of kidney[4], autoimmune[7], thyroid[8], or skin[9] in the host. Chronic inflammation tends to predispose to the development of cancer[10], and gut microbes are thought to have the ability of both inhibiting and causing cancer[11]. In addition, microbiota in the gut can mediate gut-brain crosstalk[12], which may be associated with migraines[13] and various psychiatric diseases[14]. Among all the 12 nervous system disease phenotypes, nine phenotypes have sub-attributes that show local aggregations with the significance of LAI<0.01 in the space (Supplementary Table S3). Three out of the four remaining diseases, including ADD_ADHD (Attention Deficit Disorder and Attention Deficit Hyperactivity Disorder), ALZHEIMERS, and EPILEPSY_OR_SEIZURE_DISORDER, showed significant aggregations (LAI<0.1). The relationship between the nervous system diseases, such as bulimia nervosa[15] and Post-traumatic stress disorder (PTSD)[16], and gut microbes has been relatively well studied. This also proves the validity of this paper in using the LAI to identify the correlation between phenotypes and gut microbiomes in sample space.

Among the above significantly locally aggregated phenotypes, the vast majority of locally aggregated sub-attributes are of control samples, such as “I do not have this condition”, “No”, “Never”, or “Rarely”. They are called “health sub-attribute” is this paper because the individuals in these sub-attributes are “healthy” from the perspective of specific diseases (Supplementary Tables S2 and S3). Among the disease-related sub-attributes with over 20 samples, more than 70% (40 out of 54) had LAI values over 0.3, and only 14.8% (8 out of 54) showed local aggregations (LAI < 0.1). The results of the control sub-attributes were greatly different—about 80% (26 out of 32) of

the control sub-attributes showed local aggregations, and the LAI values were all less than 0.05 (Supplementary Table S2).

This study focused on seven food-related allergies and seven non-food allergy phenotypes. The statistical results of the LAI (Supplementary Table S2) showed that, among the seven food-related allergies, five showed significant local aggregations, including GLUTEN, ALLERGIC_TO_TREE_NUTS, ALLERGIC_TO_PEANUTS, ALLERGIC_TO_I_HAVE_NO_FOOD_ALLERGIES_THAT_I_KNOW_OF, and ALLERGIC_TO_UNSPECIFIED). Among the non-food allergies, none of the other six allergies, except SEASONAL_ALLERGIES, showed significant local aggregation. The allergies to beestings, poison, ivy, oak, pet dander, and penicillin were not significantly related to gut microbes. Among the locally aggregated allergic phenotypes, local aggregations also mostly occurred in health sub-attributes.

3. Dietary characteristics and “reference daily diet style”

Among the 41 dietary habit-related characteristics, 34 showed significant local aggregations (LAI<0.1, Supplementary Table S2). Eight characteristics related to alcohol consumption demonstrated the changes in gut microbes caused by alcohol. Both ALCOHOL_FREQUENCY and DRINKS_PER_SESSION showed local aggregations, and among the six alcohol-type characteristics, the sub-attribute “Yes” of ALCOHOL_TYPES_WHITE_WINE and ALCOHOL_TYPES_BEERCIDER, as well as the sub-attribute “No” of ALCOHOL_TYPES_UNSPECIFIED showed local aggregations. The LAI values of the sub-attribute “Yes” of ALCOHOL_TYPES_SPIRITSHARD_ALCOHOL, ALCOHOL_TYPES_SOUR_BEERS, and ALCOHOL_TYPES_RED_WINE were not greater than 0.2. This may mean that specific types of alcohol tend to have relatively consistent effects on the gut microbes of different individuals. All of the nine specialized diet-related characteristics showed local aggregations. Specifically, the sub-attribute “Yes” of SPECIALIZED_DIET_I_DO_NOT_EAT_A_SPECIALIZED_DIET was locally aggregated with a significance of LAI<0.001, and the sub-attribute “No” of the remaining 8 specialized diet related characteristics, such as exclude dairy, exclude nightshades, raw food diet, showed local aggregations. Only EXCLUDE_REFINED_SUGARS saw local aggregations of individuals in both the “Yes” and “No” sub-attributes. This shows that for different individuals influenced by the same kind of specialized diet, the direction of change in gut microbiome may be different. In addition to the characteristics related to alcohol consumption and specialized diets, there were 24 characteristics of daily dietary habits, 20 of which showed significant aggregations (Supplementary Table S2). Although compared with disease-related phenotypes, it is difficult to judge how a diet-related characteristic can be “healthier”, most of the sub-attributes with significant local aggregations found in this study are highly praised “healthy dietary habits” in some way. In view of this, this study may provide a “reference daily diet style”, such as eating meat and eggs three to five times a week, including at least once or twice eating a whole egg, and reducing the frequency of eating red meat; drinking milk or eating cheese every day, and frequently consuming some milk substitutes such as grain milk and nut milk; eating vegetables every day, and consuming more than 10 kinds of vegetables per week; eating fewer sweets and do avoiding sugared beverages; cooking at home with olive oil every day (preferred), occasionally eating prepared meals (though it is not recommended to eat ready-to-eat meals every day, it seems that there is no problem to eat such meals occasionally); occasionally drinking a liter of water per day and eating some fruits, seafood, and whole grain. In this list, ARTIFICIAL_SWEETENERS is a special characteristic as individuals showed significant local aggregations regardless of the frequency of intake, whether low (“Never”, “Rarely”) or high (“Regularly”). Artificial sweeteners have been regarded as a boon for diabetics. In recent years, however, an increasing number of studies have shown that excessive intake of artificial sweeteners can lead to metabolic problems and obesity[17], and can also affect gut microbes[18], which corroborates the local aggregations of ARTIFICIAL_SWEETENERS in a variety of sub-attributes found in this study.

Supplementary Figures

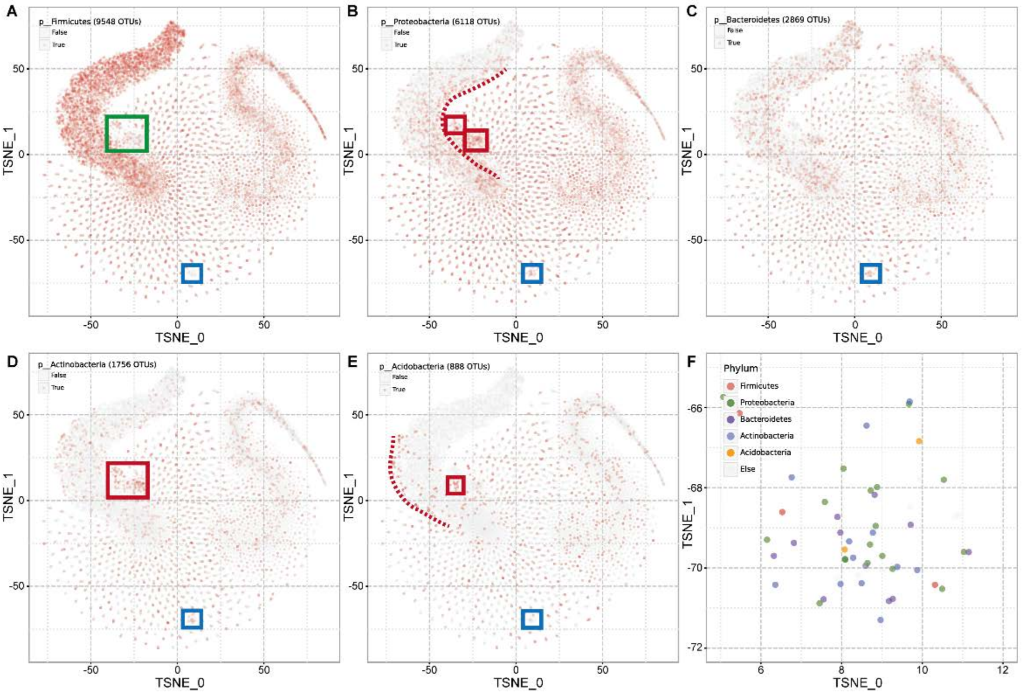

Figure S1. Distributions of the five phyla containing the largest number of OTUs in global view (A-E) and the cluster in the fourth quadrant (F).

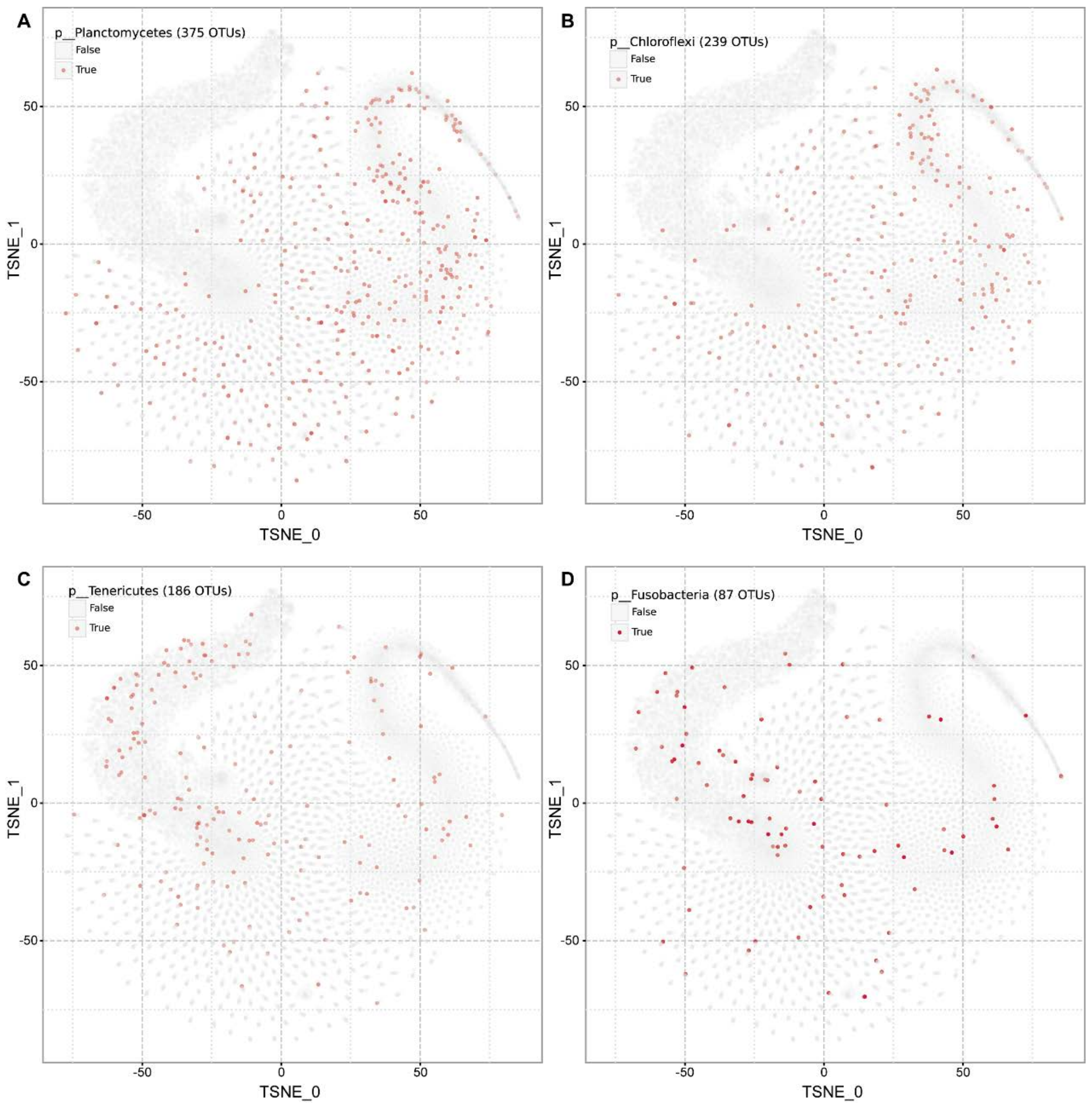

Figure S2. Global distribution of OTUs from phylum Planctomycetes (A), Chloroflexi (B), Tenericutes (C) and Fusobacteria (D).

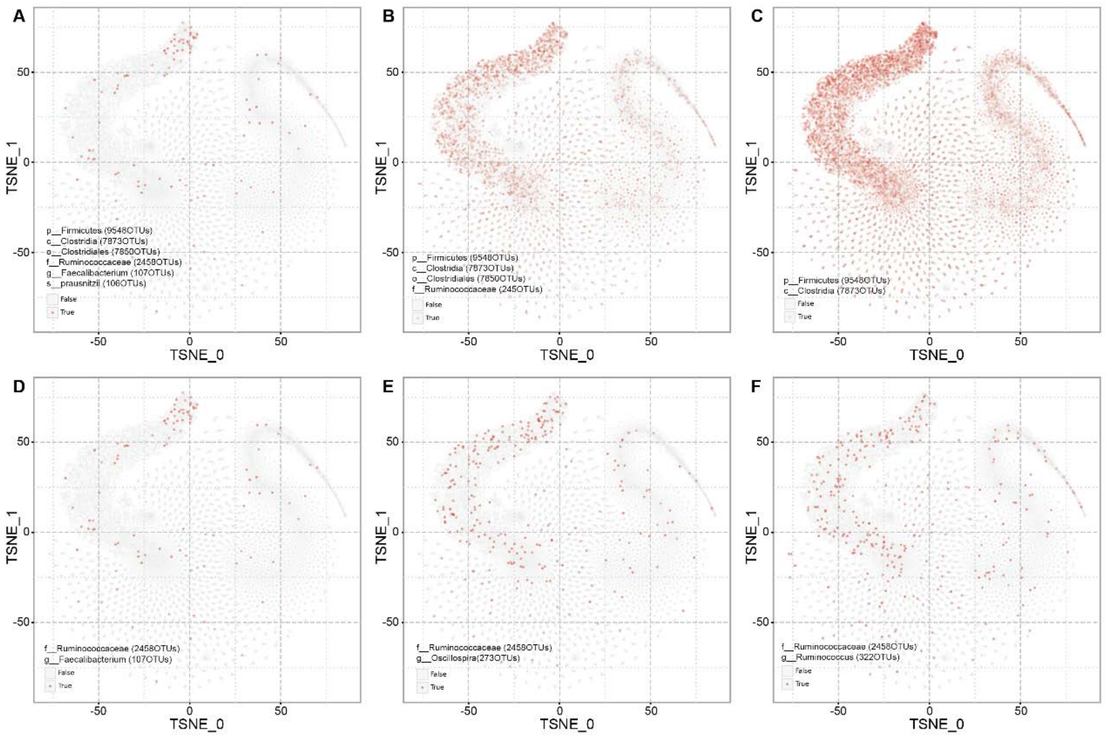

Figure S3. OTU distributions of different taxa and on different taxonomy level.

A. OTU distribution of species *F. prausnitzii*. **B,C**. The family (B) and class (C) that *F. prausnitzii* belongs to. **D-F**. The distributions of OTUs from Faecalibacterium's three main genera.

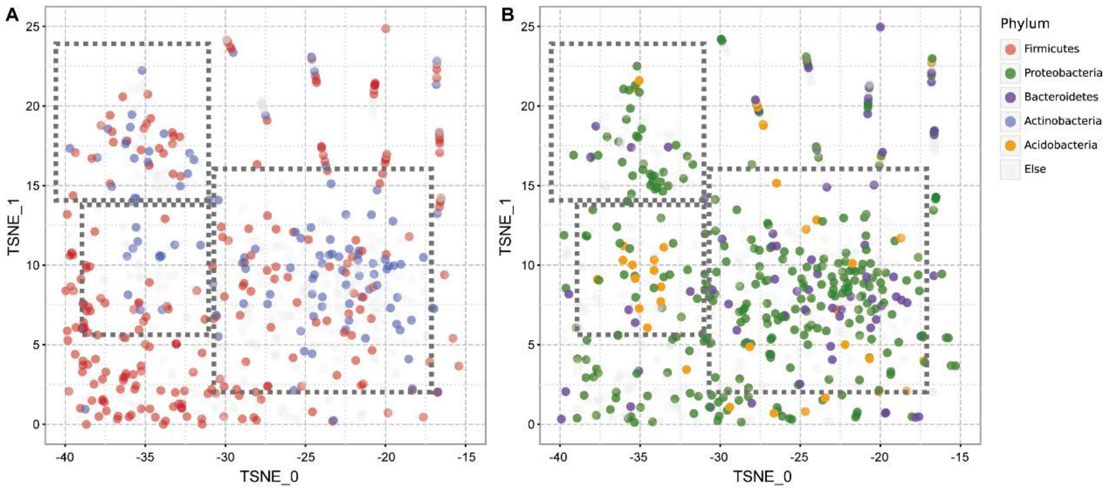

Figure S4. The region of the three clusters near left croissant.

A. OTU distribution of Firmicutes and Proteobacteria. **B.** OTU distribution of Bacteroidetes, Actinobacteria, and Acidobacteria.

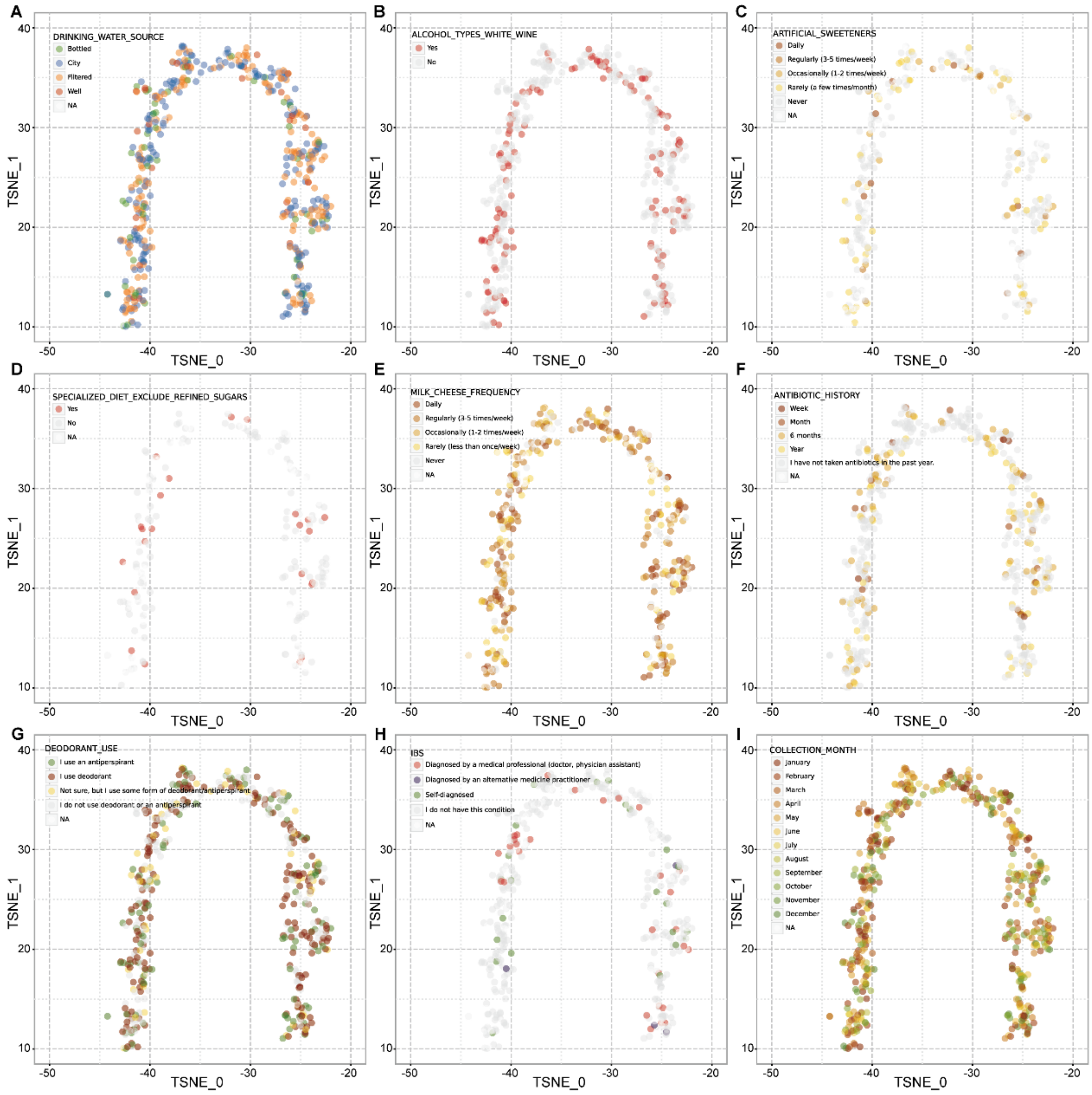

Figure S5. Distribution of different sub-attributes of nine attributes in a same example region.

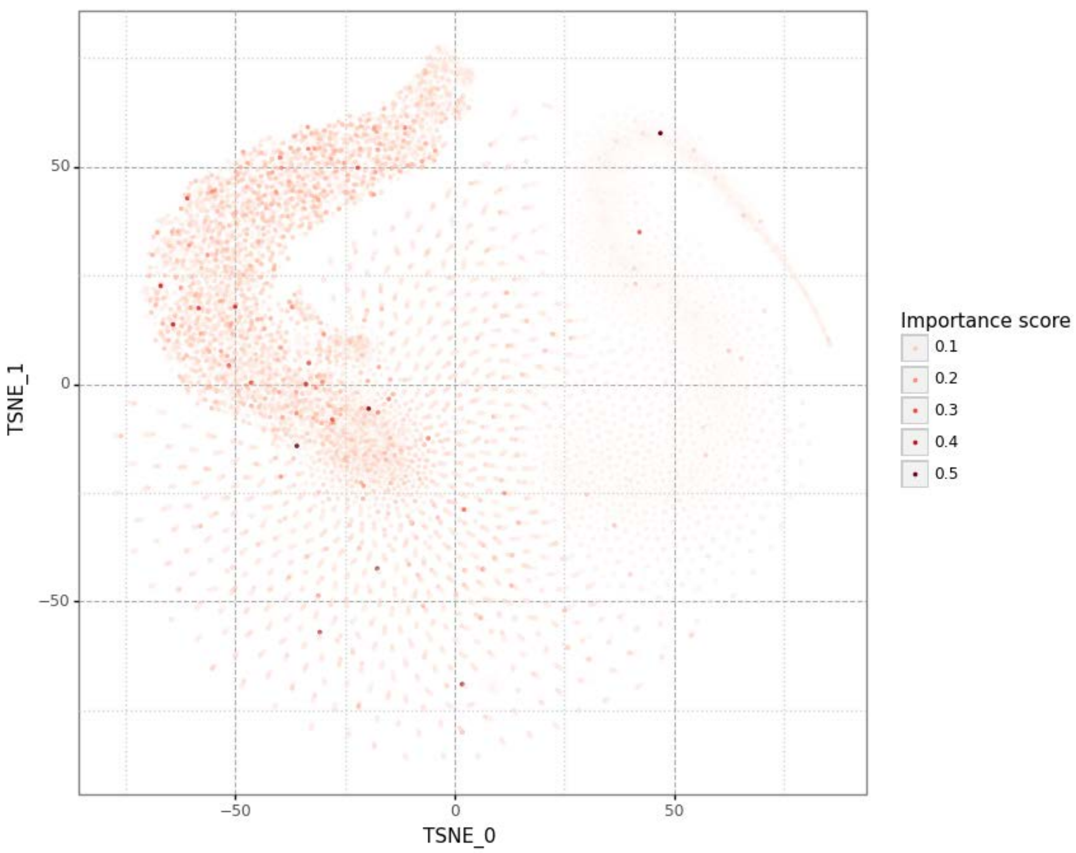

Figure S6. The importance score of OTUs for the IBD_DIAGNOSIS_REFINED classification task.

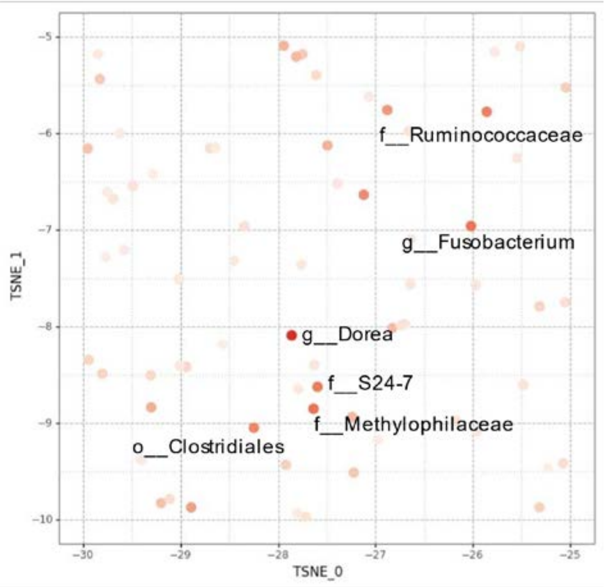

Figure S7. Zoom in for Supplementary Fig. S6.

Supplementary References

[1] VAN ROSSUM T, FERRETTI P, MAISTRENKO O M, et al. Diversity within Species: Interpreting Strains in Microbiomes[J]. Nature Reviews Microbiology, 2020, 18(9): 491–506. DOI:10.1038/s41579-020-0368-1.

[2] YOSHIDA N, YAMASHITA T, HIRATA K. Gut Microbiome and Cardiovascular Diseases[J]. Diseases, 2018, 6(3): 56. DOI:10.3390/diseases6030056.

[3] AHMADMEHRABI S, TANG W H W. Gut Microbiome and Its Role in Cardiovascular Diseases[J]. Current Opinion in Cardiology, 2017, 32(6): 761–766. DOI:10.1097/HCO.0000000000000445.

[4] WEHEDY E, SHATAT I F, AL KHODOR S. The Human Microbiome in Chronic Kidney Disease: A Double-Edged Sword[J]. Frontiers in Medicine, 2022, 8. DOI:10.3389/fmed.2021.790783.

[5] EBERT T, PAINER J, BERGMAN P, et al. Insights in the Regulation of Trimetylamine N-Oxide Production Using a Comparative Biomimetic Approach Suggest a Metabolic Switch in Hibernating Bears[J]. Scientific Reports, 2020, 10(1). DOI:10.1038/s41598-020-76346-1.

[6] EL-DEEB O S, ATEF M M, HAFEZ Y M. The Interplay between Microbiota-Dependent Metabolite Trimethylamine N-Oxide, Transforming Growth Factor β/SMAD Signaling and Inflammasome Activation in Chronic Kidney Disease Patients: A New Mechanistic Perspective[J]. Journal of Cellular Biochemistry, 2019, 120(9): 14476–14485. DOI:10.1002/jcb.28707.

[7] DE LUCA F, SHOENFELD Y. The Microbiome in Autoimmune Diseases[J]. Clinical and Experimental Immunology, 2019, 195(1): 74–85. DOI:10.1111/cei.13158.

[8] KNEZEVIC J, STARCHL C, BERISHA A T, et al. Thyroid-Gut-Axis: How Does the Microbiota Influence Thyroid Function?[J]. Nutrients, 2020, 12(6): 1–16. DOI:10.3390/nu12061769.

[9] MAHMUD M R, AKTER S, TAMANNA S K, et al. Impact of Gut Microbiome on Skin Health: Gut-Skin Axis Observed through the Lenses of Therapeutics and Skin Diseases[J]. Gut Microbes, 2022, 14(1). DOI:10.1080/19490976.2022.2096995.

[10] GRETEN F R, GRIVENNIKOV S I. Inflammation and Cancer: Triggers, Mechanisms, and Consequences[J]. Immunity, 2019, 51(1): 27–41. DOI:10.1016/j.immuni.2019.06.025.

[11] VIVARELLI S, SALEMI R, CANDIDO S, et al. Gut Microbiota and Cancer: From Pathogenesis to Therapy[J]. Cancers, 2019, 11(1). DOI:10.3390/cancers11010038.

[12] RIEDER R, WISNIEWSKI P J, ALDERMAN B L, et al. Microbes and Mental Health: A Review[J]. Brain, Behavior, and Immunity, 2017, 66: 9–17. DOI:10.1016/j.bbi.2017.01.016.

[13] CRAWFORD J, LIU S, TAO F. Gut Microbiota and Migraine.[J/OL]. Neurobiology of Pain (Cambridge, Mass.), 2022, 11: 100090. http://www.ncbi.nlm.nih.gov/pubmed/35464185%0Ahttp://www.pubmedcentral.nih.gov/articlerender.fcgi?artid=PMC9018445. DOI:10.1016/j.ynpai.2022.100090.

[14] BUTLER M I, MÖRKL S, SANDHU K V., et al. The Gut Microbiome and Mental Health: What Should We Tell Our Patients?: Le Microbiote Intestinal et La Santé Mentale : Que

Devrions-Nous Dire à Nos Patients?[J]. Canadian Journal of Psychiatry, 2019, 64(11): 747–760. DOI:10.1177/0706743719874168.

[15] HERMAN A, BAJAKA A. The Role of the Intestinal Microbiota in Eating Disorders – Bulimia Nervosa and Binge Eating Disorder[J]. Psychiatry Research, 2021, 300. DOI:10.1016/j.psychres.2021.113923.

[16] LECLERCQ S, FORSYTHE P, BIENENSTOCK J. Posttraumatic Stress Disorder: Does the Gut Microbiome Hold the Key?[J]. Canadian Journal of Psychiatry, 2016, 61(4): 204–213. DOI:10.1177/0706743716635535.

[17] PEARLMAN M, OBERT J, CASEY L. The Association Between Artificial Sweeteners and Obesity[J]. Current Gastroenterology Reports, 2017, 19(12). DOI:10.1007/s11894-017-0602-9.

[18] SUEZ J, KOREM T, ZEEVI D, et al. Artificial Sweeteners Induce Glucose Intolerance by Altering the Gut Microbiota[J]. Obstetrical and Gynecological Survey, 2015, 70(1): 31–32. DOI:10.1097/01.ogx.0000460711.58331.94.
